# Bone marrow adipogenic lineage precursors are the major regulator of bone resorption in adult mice

**DOI:** 10.1038/s41413-025-00405-4

**Published:** 2025-03-19

**Authors:** Jiawei Lu, Qi He, Huan Wang, Lutian Yao, Michael Duffy, Hanli Guo, Corben Braun, Yilu Zhou, Qiushi Liang, Yuewei Lin, Shovik Bandyopadhyay, Kai Tan, Yongwen Choi, X. Sherry Liu, Ling Qin

**Affiliations:** 1 Department of Orthopaedic Surgery, Perelman School of Medicine, University of Pennsylvania, Philadelphia, PA 19104 USA; 2 Department of Spine Surgery, Shanghai East Hospital, School of Medicine, Tongji University, Shanghai, 200092 China; 3 Department of Pediatrics, Perelman School of Medicine, University of Pennsylvania, Philadelphia, PA 19104 USA; 4 Center for Childhood Cancer Research, The Children’s Hospital of Philadelphia, Philadelphia, PA 19104 USA; 5 Department of Pathology and Laboratory Medicine, Perelman School of Medicine, University of Pennsylvania, Philadelphia, PA 19104 USA

**Keywords:** Osteoporosis, Osteopetrosis

## Abstract

Bone resorption by osteoclasts is a critical step in bone remodeling, a process important for maintaining bone homeostasis and repairing injured bone. We previously identified a bone marrow mesenchymal subpopulation, marrow adipogenic lineage precursors (MALPs), and showed that its production of RANKL stimulates bone resorption in young mice using *Adipoq-Cre*. To exclude developmental defects and to investigate the role of MALPs-derived RANKL in adult bone, we generated inducible reporter mice (*Adipoq-CreER Tomato*) and RANKL deficient mice (*Adipoq-CreER RANKLflox/flox, iCKO*). Single cell-RNA sequencing data analysis and lineage tracing revealed that Adipoq^+^ cells contain not only MALPs but also some mesenchymal progenitors capable of osteogenic differentiation. In situ hybridization showed that *RANKL* mRNA is only detected in MALPs, but not in osteogenic cells. RANKL deficiency in MALPs induced at 3 months of age rapidly increased trabecular bone mass in long bones as well as vertebrae due to diminished bone resorption but had no effect on the cortical bone. Ovariectomy (OVX) induced trabecular bone loss at both sites. RANKL depletion either before OVX or at 6 weeks post OVX protected and restored trabecular bone mass. Furthermore, bone healing after drill-hole injury was delayed in *iCKO* mice. Together, our findings demonstrate that MALPs play a dominant role in controlling trabecular bone resorption and that RANKL from MALPs is essential for trabecular bone turnover in adult bone homeostasis, postmenopausal bone loss, and injury repair.

## Introduction

Bone is critical for protecting internal organs, supporting the body, allowing movement, as well as hosting hematopoiesis. To maintain its essential structure and functions, bone undergoes continuous remodeling—a cyclic process involving osteoclastic bone resorption and osteoblastic/osteocytic bone formation.^1^ In healthy adults, this remodeling process is precisely balanced to preserve normal bone mass. In aged and diseased populations, this balance is shifted towards resorption rather than formation, leading to osteoporosis characterized by low bone mass, deteriorated bone structure, and high risk of fracture.^1^ Following injuries, bone remodeling is a crucial process that facilitates the bridging of the fracture gap.^2^

One important aspect of bone remodeling is to initiate osteoclast formation at the trabecular or cortical bone surface for bone resorption. Descended from myeloid progenitors of the hematopoietic lineage, osteoclasts are highly specific, large multinucleated, and phagocytic cells secreting acid and catalytic enzymes to demineralize and degrade collagen rich bone extracellular matrix (ECM).^3^ For a long time, they were thought to be short-lived cells that rapidly undergo apoptosis following their fusion from mononuclear progenitors.^4^ Recent in vivo research using advanced intravital imaging techniques discovered that they are actually long-lived cells constantly undergoing recycling through fission and fusion mechanism.^5–7^ Instead of apoptosis, the fission products, osteomorphs, can refuse among each other or with existing osteoclasts to extend the longevity of osteoclasts.^6^

Past research has pointed out two cytokines as the most important regulatory factors for osteoclast formation and function: colony-stimulating factor (Csf1) and receptor activator of nuclear factor kappa Β ligand (RANKL). The former one promotes the proliferation of osteoclast precursors and their expression of receptor activator of nuclear factor kappa Β (RANK), a RANKL receptor.^8^ The latter one is the predominant factor that drives the differentiation of osteoclast precursors into mature osteoclasts.^9^ It also stimulates osteoclasts to migrate and undergo cycles of fusion and fission in vivo.^6^ Encoded by *Tnfsf11*, RANKL belongs to tumor necrosis factor (TNF) superfamily and exists in two forms: membrane-bound and soluble.^10^ Upon binding to RANK, it initiates the transcription of a cascade of osteoclast specific genes via up-regulating the expression of a master transcription factor nuclear factor of activated T-cells, cytoplasmic 1 (NFATc1).^11^ Early research identified osteogenic cells, particularly bone matrix-embedded osteocytes, are the major source of RANKL that regulates osteoclast formation.^12–14^ This finding fits well with the concept of bone remodeling as it emphasizes the crosstalk between bone forming and resorbing cells.

In the past several years, the application of advanced single cell transcriptomics approaches to bone research greatly expanded our knowledge about cellular components of bone tissue and their transcriptome profiles. In particular, single cell RNA-sequencing (scRNA-seq) of mesenchymal lineage cells revealed a unique mesenchymal subpopulation that highly expresses adipogenic markers, including *Pparg, Cebpa, Lpl, Adipoq*, etc., but does not contain lipid droplets.^15,16^ Since they are precursors for mature adipocytes, we termed them marrow adipogenic lineage precursors (MALP).^15,17^ Other groups also identified similar cell types and named them Adipo-Cxcl12-abundant-reticular (Adipo-CAR) cells or marrow Adipoq^+^ cells (MACs).^16,18^ Interestingly, scRNA-seq suggested that MALPs, but not osteoblasts nor osteocytes, are the major source of RANKL and Csf1 in bone.^19,20^ Subsequent studies from our group and other groups confirmed that specific deletion of either factor in adipogenic lineage cells results in a drastically elevated trabecular bone mass due to diminished osteoclasts.^19–22^

However, those studies have limitations because they used a constitutive Cre, *Adipoq-Cre*, to examine the action of MALPs-derived factors. MALPs emerge in mouse bone right after birth.^23^ While those studies analyzed mice up to 6 months of age, it is possible that changes in adult bone is due to developmental defect. In addition, since *Adipoq-Cre* also labels bone forming cells in adult mice, Adipoq^+^ cells were regarded as bipotent bone marrow skeletal stem/progenitor cells.^24^ Hence, we cannot exclude the possibility that osteoclast regulatory factors are also depleted in osteoblasts and osteocytes in those studies. To circumvent these limitations, in this study, we first performed a lineage tracing experiment using inducible *Adipoq-CreER Tomato* (*AdipoqER/Td*) mice to delineate the relationship between MALPs and Adipoq^+^ cells. Next, we adopted RNA fluorescence in situ hybridization (FISH) to identify *RANKL*-expressing cells in vivo. Finally, we constructed inducible RANKL deficient mice using *Adipoq-CreER* and examined their adult bones under normal, estrogen depletion, and injury repair conditions. Our data revealed MALPs as the main source of RANKL in adult mice.

## Results

### Adipoq^+^ cells contain not only MALPs but also some bipotent mesenchymal progenitors

**Fig. 1:**
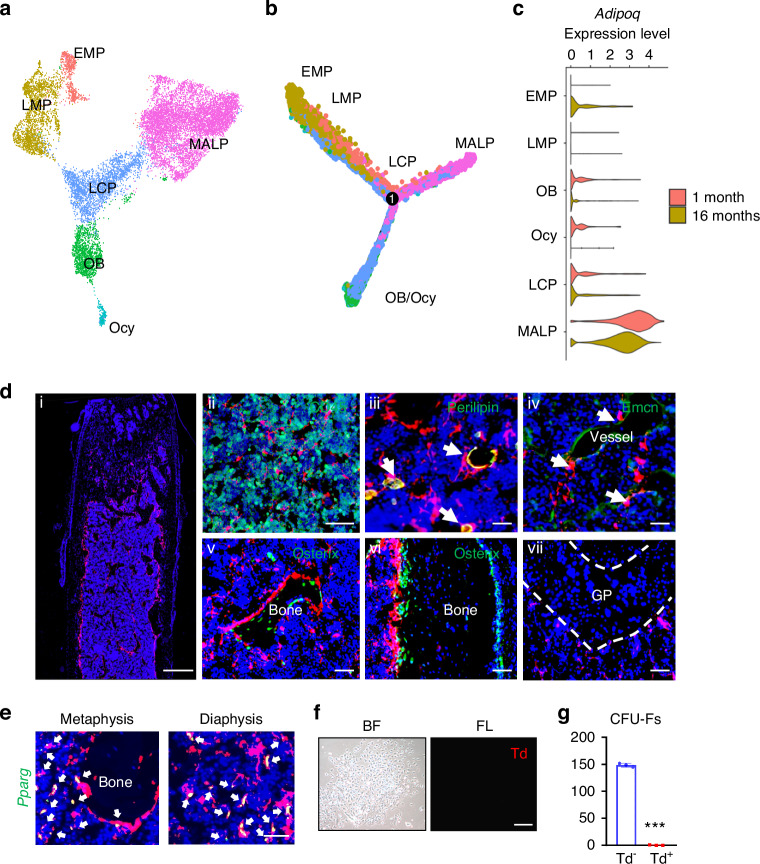
*Adipoq* labels MALPs in adult mice. **a** The integrated scRNA-seq dataset of sorted bone marrow Td^+^ cells from 1 and 16-month-old *Col2-Cre Td* mice. The Uniform Manifold Approximation and Projection (UMAP) plot is presented to show cell clustering. EMP early mesenchymal progenitor, LMP late mesenchymal progenitor, LCP lineage committed progenitor, OB osteoblast, Ocy osteocyte, MALP marrow adipogenic lineage precursor. **b** Monocle trajectory plot of bone marrow mesenchymal lineage cells. Cells are labeled according to their Seurat clusters. **c** Violin plot of *Adipoq* in bone marrow mesenchymal cells in young and old mice. **d** Representative fluorescent images of *AdipoqER/Td* mouse femur reveal many bone marrow Td^+^ cells. Mice at 3 months of age received Tam injections for 3 days and their bones were harvested at day 7. (i) A low magnification image of a distal femur. Scale bar = 500 μm. (ii–vii) At a high magnification, Td labels CD45- stromal cells (ii), Perilipin^+^ adipocytes (arrows, iii), and pericytes (arrows, iv), but does not label osteoblasts and osteocytes (v, vi) and growth plate (GP) chondrocytes (vii). Scale bar = 50 μm. **e** Fluorescent images of *AdipoqER/Td* mouse bone marrow stained for *Pparg* mRNA by RNA FISH. Scale bar=20 μm. **f** CFU-F assay of bone marrow cells from *AdipoqER/Td* mice shows that all CFU-F colonies are made of Td- cells. BF: brightfield; FL: fluorescent light. Scale bar=50 μm. **g** Quantification of Td^+^ and Td^−^ CFU-F colonies formed from 3 million bone marrow cells. ***P < 0.001 vs Td- CFU-Fs, *n* = 3 mice

We previously used *Adipoq-Cre* to study MALPs in vivo. To examine whether Adipoq^+^ cells contain other progenitors, we integrated scRNA-seq datasets of bone marrow mesenchymal cells from 1- and 16-month-old mice we previously reported (Fig. 1a).^15^ Cell clusters were annotated by their marker gene expression (Fig. S1A). Pseudotime trajectory analysis revealed that early mesenchymal progenitors (EMPs) give rise to late mesenchymal progenitors (LMPs) and lineage committed progenitors (LCPs), which are then differentiated into either adipogenic lineage (MALPs) or osteogenic lineage (osteoblasts and osteocytes) (Fig. 1b). Violin plots clearly showed that while *Adipoq* is highly expressed in MALPs, it is also expressed in LCPs followed by osteoblasts and osteocytes in 1 month dataset at very low levels (Fig. 1c). Interestingly, EMPs express *Adipoq* and other MALP markers at 16 months of age, albeit their expression levels were much lower than those in MALPs (Fig. 1c, Fig. S1B).

To analyze Adipoq^+^ cells in adult mice, we generated inducible Td reporter mice driven by *Adipoq-CreER*. These mice, *AdipoqER/Td*, at 3 months of age received daily Tamoxifen (Tam) injections from day 1 to 3. At day 7, many Td^+^ cells were observed inside the long bone (Fig. 1d(i)). Within the metaphysis region, Td^+^ cells were made of 73.5% ± 1.0% Cd45- stromal cells, 2.7% ± 0.2% Perilipin^+^ adipocytes, 9.0% ± 0.4% pericytes, and 14.8% ± 1.2% bone lining cells [Fig. 1d(ii–v), *n* = 5 mice]. Although some bone surface lining cells were also Td ^+^ , they did not express Osterix, an osteogenic cell marker [Fig. 1d(v, vi)]. Particularly at the endocortical bone surface, we observed a lot of Td^+^ cells in the close proximity to Osterix^+^ osteoblasts. Td did not label chondrocytes, osteocytes or periosteal cells [Fig. 1d(vi, vii)]. In addition, in situ staining of *Pparg*, the master transcriptional factor for adipogenic differentiation^25^ and another marker for MALPs,^15^ showed that it is only expressed in Td^+^ cells (Fig. 1e). These data indicate that *Adipoq-CreER* targets MALPs, but not bone forming cells (osteoblasts and osteocytes). Furthermore, CFU-F assay showed that almost all CFU-F colonies are Td- (Fig. 1f, g), suggesting that Adipoq^+^ cells lack the proliferation ability required by early progenitors.

**Fig. 2:**
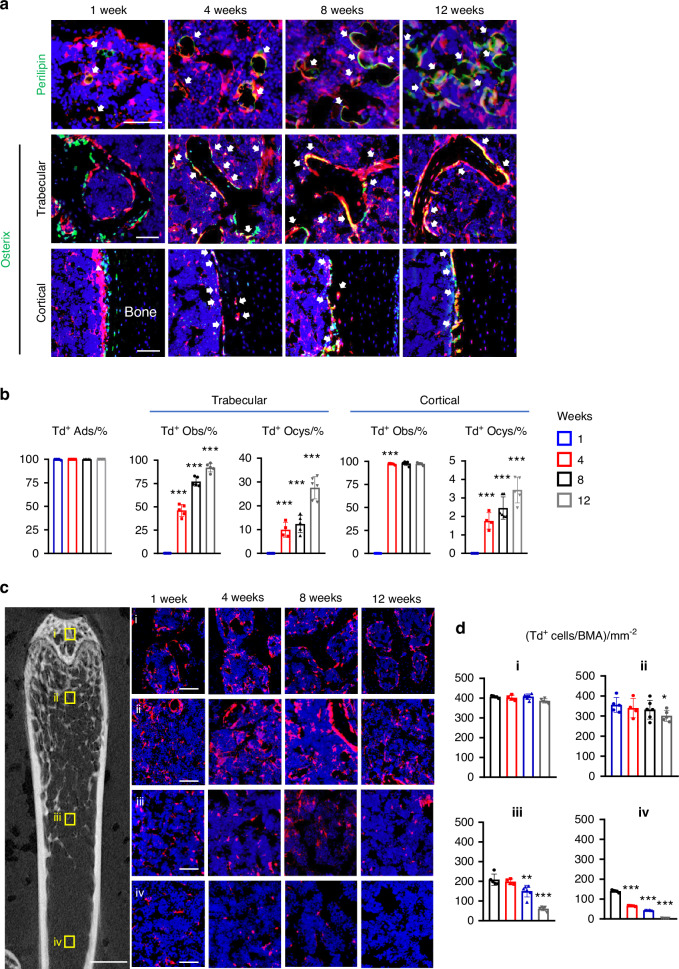
*Adipoq* also labels some late, bipotent mesenchymal progenitors. **a** Fluorescent images of *AdipoqER/Td* mouse bone marrow stained for Perilipin or Osterix protein. Mice at 3 months of age received Tam injections for 3 days and their femurs were harvested at 1, 4, 8 and 12 weeks later. In the top panel, arrows point to mature adipocytes. In the middle (trabecular bone) and bottom (cortical bone) panels, arrows point to Osterix^+^Td^+^ cells. Scale bar = 50 μm. **b** Percentages of Td^+^ cells in adipocytes (ADs), osteoblasts (OBs), and osteocytes (Ocys) were quantified over the tracing period. *n* = 4–6 mice/time point. **c** Fluorescent images of *AdipoqER/Td* bone marrow to show location-dependent change of Td^+^ cells during tracing. The left panel is a representative 2D microCT image of femur to show 4 areas for quantification. Scale bar = 1 mm. Their corresponding areas in the fluorescent images during the tracing period are shown at the right. i: subchondral bone; ii: top metaphysis; iii: bottom metaphysis; iv: diaphysis (scale bar = 50 μm). **d** Quantification of Td^+^ cells per bone marrow area (BMA) in 4 areas. *P < 0.05; **P < 0.01; ***P < 0.001 vs 1 week, *n* = 5 mice/time point

To determine the fate of Adipoq^+^ cells, we harvested long bones of *AdipoqER/Td* mice at 1, 4, 8 and 12 weeks post the first Tam injection for lineage tracing experiment (Fig. 2a, b). Perilipin staining revealed that Td labels nearly all mature adipocytes throughout the tracing period. On the contrary, Td gradually labeled osteoblasts and osteocytes over time. While no Td^+^ osteoblasts and osteocytes were detected at 1 week in both trabecular and cortical bone, the percentages of Td^+^ osteoblasts increased to 46.1%, 77.1% and 92.1% and the percentages of Td^+^ osteocytes increased to 9.9%, 12.4% and 27.6% in the trabecular bone at 4, 8 and 12 weeks, respectively. In the cortical bone, almost all endosteal osteoblasts became Td^+^ after 4 weeks but few osteocytes (3.4%) became Td^+^ even after 12 weeks of tracing. These data suggest that Adipoq^+^ cells contain not only committed adipo-lineage cells but also uncommitted mesenchymal progenitors capable of osteogenic differentiation.

We noticed that Td^+^ cells are not evenly distributed through the bone marrow. Thus, we counted them at four anatomic sites: subchondral bone, top metaphysis (region close to the growth plate), bottom metaphysis (region distal to the growth plate), and diaphysis (Fig. 2c). Interestingly, we found that the density of bone marrow Td^+^ cells (excluding bone surface and embedded cells) is much lower in the midshaft region compared to the trabecular bone region. During the 3-month tracing period, Td^+^ cells in the area with high trabecular bone volume (subchondral bone and top metaphysis) remained unaltered, but Td^+^ cells in the area with low trabecular bone volume (bottom metaphysis and diaphysis) decreased significantly (Fig. 2c, d). These data indicate that mesenchymal progenitors labeled by *Adipoq-CreER* are not early-stage progenitors with self-renewal ability. Together with CFU-F result, our lineage tracing data indicate that Adipoq^+^ cells likely include some late, bipotent mesenchymal progenitors, possibly LCPs identified in scRNA-seq.

### MALPs are the major producer of osteoclast regulator factors in adult bone

**Fig. 3:**
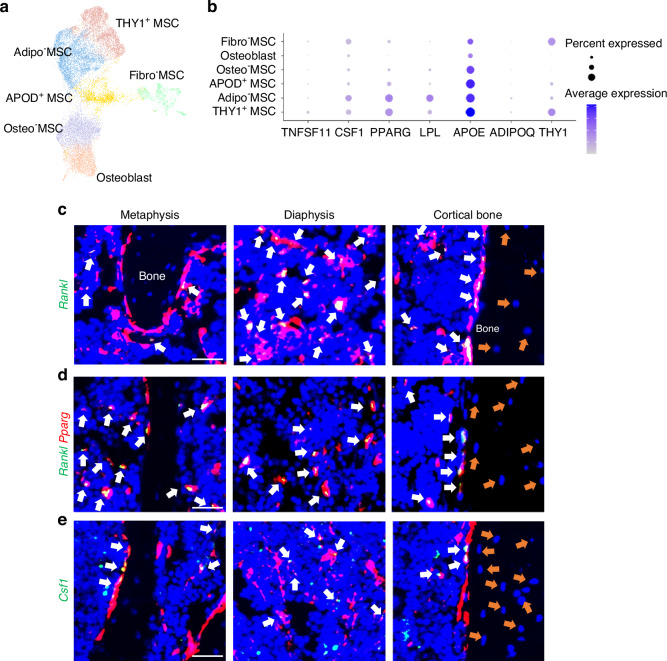
MALPs are the major source of osteoclast regulatory factors in bone marrow. **a** UMAP plot of mesenchymal subpopulations in human bone marrow. Bone samples were collected from femoral heads after hip replacement surgery. **b** Dot plot of *TNFSF11*, *CSF1*, *THY1* and adipogenic markers in mesenchymal subpopulations. **c** Fluorescent images of *AdipoqER/Td* mouse bone marrow stained for *Rankl* mRNA by RNA FISH. White arrows: *Rankl* ^+^ Td^+^ cells; orange arrows: Osteocytes. Scale bar = 20 μm. **d** Fluorescent images of bone marrow co-stained for *Pparg and Rankl* mRNA by RNA FISH. White arrows: *Rankl*^+^*Ppagr*^+^ cells; orange arrows: osteocytes. Scale bar = 20 μm. **e** Fluorescent images of *AdipoqER/Td* mouse bone marrow stained for *Csf1* mRNA by RNA FISH. White arrows: *Csf1* ^+^ Td^+^ cells; orange arrows: osteocytes. Scale bar = 20 μm

Our previous scRNA-seq of mouse bone marrow predicted that MALPs are the major producers of osteoclast regulatory factors, including RANKL and Csf1.^15^ We recently profiled bone marrow from human femoral heads.^26^ Cell clustering revealed 6 mesenchymal cell clusters: Fibro-MSC (mesenchymal stromal cell), APOD ^+^ MSC, Adipo^−^MSC, THY1 ^+^ MSC, Osteo^−^MSC and Osteoblast (Fig. 3a). Among these clusters, Adipo^−^MSC and THY1 ^+^ MSC highly expressed adipogenic genes, and a major difference between them is THY1 expression. Thus, we consider them both as human counterpart of MALPs (Fig. 3b). In line with mouse data (Fig. S2), *RANKL* (*TNFSF11*) was mainly expressed in THY1^+^ MSCs, while *CSF1* was mainly expressed in Adipo^−^ and THY1^+^ MSCs followed by Fibro^−^MSCs. Their expression was much lower in osteolineage cells than in MALPs. We also examined their expression in other bone marrow cells (Fig. S3). While *RANKL* expression was restricted to mesenchymal lineage cells, *CSF1* expression was broader, including megakaryocyte-erythroid progenitors (MEPs), erythroblasts, basophil/eosinophil/mast (Ba/Eo/Ma) cells, blood vessel cells etc. However, the highest expression was still detected in Adipo-MSCs.

To confirm this finding, we stained *Rankl* in situ on 3-month-old *AdipoqER/Td* mouse femurs harvested at day 7 after the first Tam injection. Interestingly, almost all *Rankl*-expressing cells were Td^+^ (Fig. 3c, *n* = 5 mice). Most of them resided in the metaphyseal and diaphyseal bone marrow and some were on the trabecular and cortical bone surface. Moreover, co-staining showed that *Rankl*^+^ cells were also *Pparg*^+^ cells (Fig. 3d). On the contrary, only 60.2% ± 0.8% of *Csf1*-expressing cells were Td^+^ (Fig. 3e, *n* = 5 mice). Importantly, we did not observe any *Rankl* and *Csf1* mRNA expression in osteocytes in either trabecular bone or cortical bone (Fig. 3c, e). Similarly, in human bone, *RANKL* expression was mainly detected in *PPARG*-expressing cells in the bone marrow (Fig. S4, *n* = 3), demonstrating that MALPs, but not osteogenic cells, are the major cell source of osteoclast regulatory factors.

### MALP-derived RANKL supports bone resorption in adult mice

**Fig. 4:**
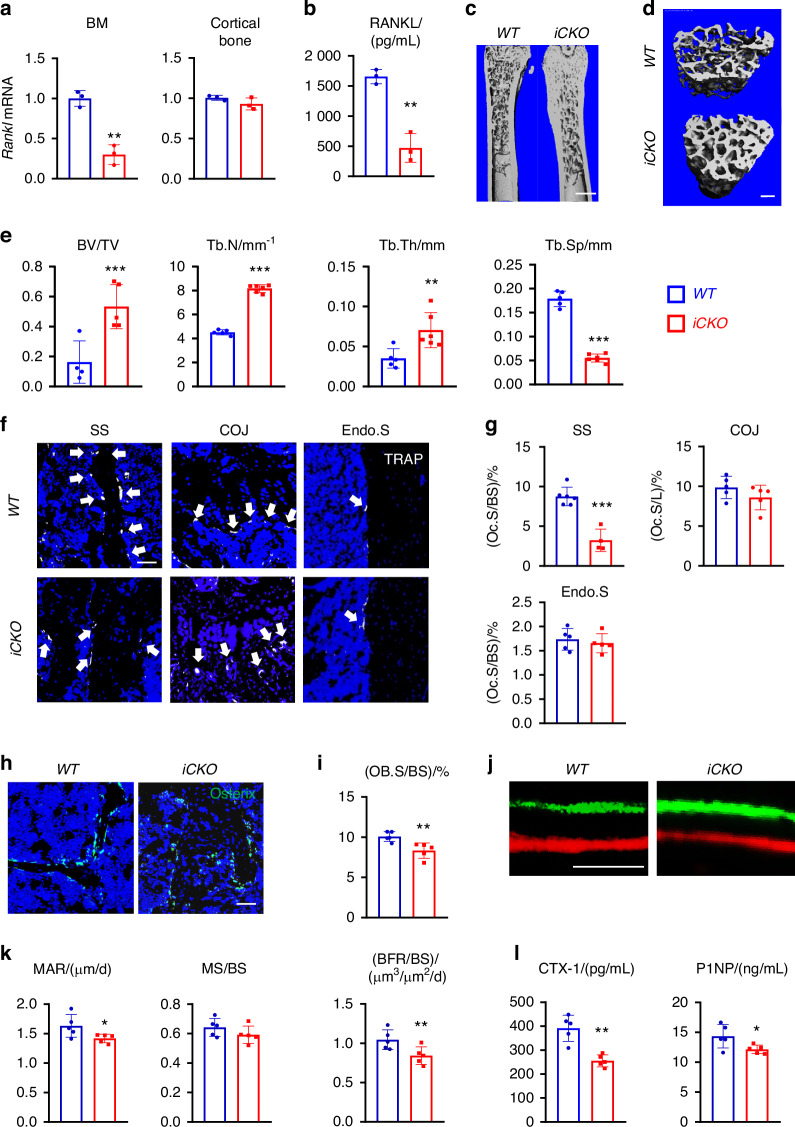
Depletion of RANKL in MALPs increases long bone trabecular bone mass in adult mice by suppressing bone resorption. **a** qRT-PCR analysis of *Rankl* mRNA in bone marrow and cortical bone from *WT* and *RANKL iCKO* mice at 4 weeks after Tam injection. Mice received Tam at 3 months of age. *n* = 3 mice/group. **b** ELISA analysis of RANKL in bone marrow from *WT* and *RANKL iCKO* mice at 2 weeks after Tam injection. Mice received Tam at 3 months of age. *n* = 3 mice/group. **c** 3D microCT reconstruction of whole femurs from *WT* and *iCKO* mice at 1 month after Tam injection. Scale bar = 1 mm. **d** 3D microCT reconstruction reveals a drastic increase of femoral trabecular bone. Scale bar = 200 µm. **e** MicroCT measurement of trabecular bone structural parameters. BV/TV bone volume fraction, Tb.N trabecular number, Tb.Th trabecular thickness, Tb.Sp trabecular separation. **f** Representative TRAP staining images show TRAP+ osteoclast (arrows) at different skeletal sites: secondary spongiosa (SS), chondro-osseous junction (COJ), and endosteal surface (Endo.S). Scale bar = 50 μm. **g** Quantification of osteoclast surface (Oc.S) at 3 skeletal sites. BS bone surface, L COJ length. **h** Representative Osterix staining of trabecular bone from *WT* and *RANKL iCKO* femurs. Scale bar = 50 μm. **i** Quantification of osteoblast surface (OB.S). **j** Representative double labeling of trabecular bone from *WT* and *iCKO* femurs. Scale bar = 20 μm. **k** Bone formation activity is quantified. MAR mineral apposition rate, MS mineralizing surface, BFR bone formation rate. **l** Serum ELISA analysis of bone resorption marker (CTX-1) and formation marker (P1NP) in *WT* and *iCKO* mice. *P < 0.05; **P < 0.01; ***P < 0.001 vs *WT*, *n* = 5–6 mice/group

To investigate the role of MALP-derived RANKL in adult bone remodeling, we constructed *Adipoq-CreER RANKL*^*flox/flox*^ (*RANKL iCKO*) mice. At 3 months of age, these mice displayed similar trabecular and cortical bone structures in femurs and vertebrae as *WT* siblings (Fig. S5). Next, we subjected both *WT* and *iCKO* mice to Tam injections for 3 days. Four weeks later, *Rankl* mRNA were reduced by 70.0% and 2 weeks later, RANKL protein amount were reduced by 71.6%, respectively, in bone marrow from *iCKO* mice but not in the cortical bone (Fig. 4a, b). This change did not alter their body weight (Fig. S6A) and longitudinal bone growth, as indicated by growth plate thickness and femoral bone length (Fig. S6B–D). Strikingly, compared to *WT* mice, *iCKO* mice exhibited a 3.3-fold increase in trabecular bone volume fraction (BV/TV), a 1.8-fold increase in trabecular number (Tb.N), a 2.0-fold increase in trabecular thickness, and a 69.7% decrease in trabecular separation (Tb.Sp) at 4 weeks post Tam injections (Fig. 4c–e). These mice gained trabecular bone mass quickly as the increase of BV/TV can be detected as early as 2 weeks post Tam injections (Fig. S7A, B). On the contrary, the cortical bone structure of *iCKO* mice was not altered (Fig. S8). Similar massive bone gain phenotype was also observed in vertebrae (Fig. S9).

We next performed bone histomorphometry to uncover the cellular changes. TRAP staining revealed that osteoclasts are greatly reduced by 44.7% and 63.0% at the trabecular bone surface at 2 and 4 weeks post Tam injections, respectively, but not changed at the chondro-osseous junction (COJ) between growth plate and primary spongiosa and endosteal bone surface (Fig. 4f, g, Fig. S7C, D). Meanwhile, osteoblasts (Osterix^+^ bone surface cells) was decreased by 12.5% and 17.4%, respectively (Fig. 4h, i, Fig. S7E, F) and their activity was also significantly reduced (Fig. 4j, k). Serum chemistry confirmed those changes, showing a 34.7% reduction in bone resorption marker CTX-1 and a 14.2% reduction in bone formation marker P1NP (Fig. 4l). Overall, these data show that MALP-derived RANKL is important for maintaining bone resorption in adult mice.

RANKL not only regulates bone metabolism but also hematopoietic cells.^9^ Since RANKL is expressed in MALPs that are distributed throughout the bone marrow, we examined hematopoietic cells in *iCKO* mice. However, flow analysis did not detect any changes in hematopoietic components in the bone marrow or peripheral blood (Fig. S10A, B). Their spleen weight was not altered either (Fig. S10C), suggesting that hematopoiesis is normal in *iCKO* mice.

### RANKL depletion in MALPs attenuates ovariectomy (OVX)-induced bone loss

**Fig. 5:**
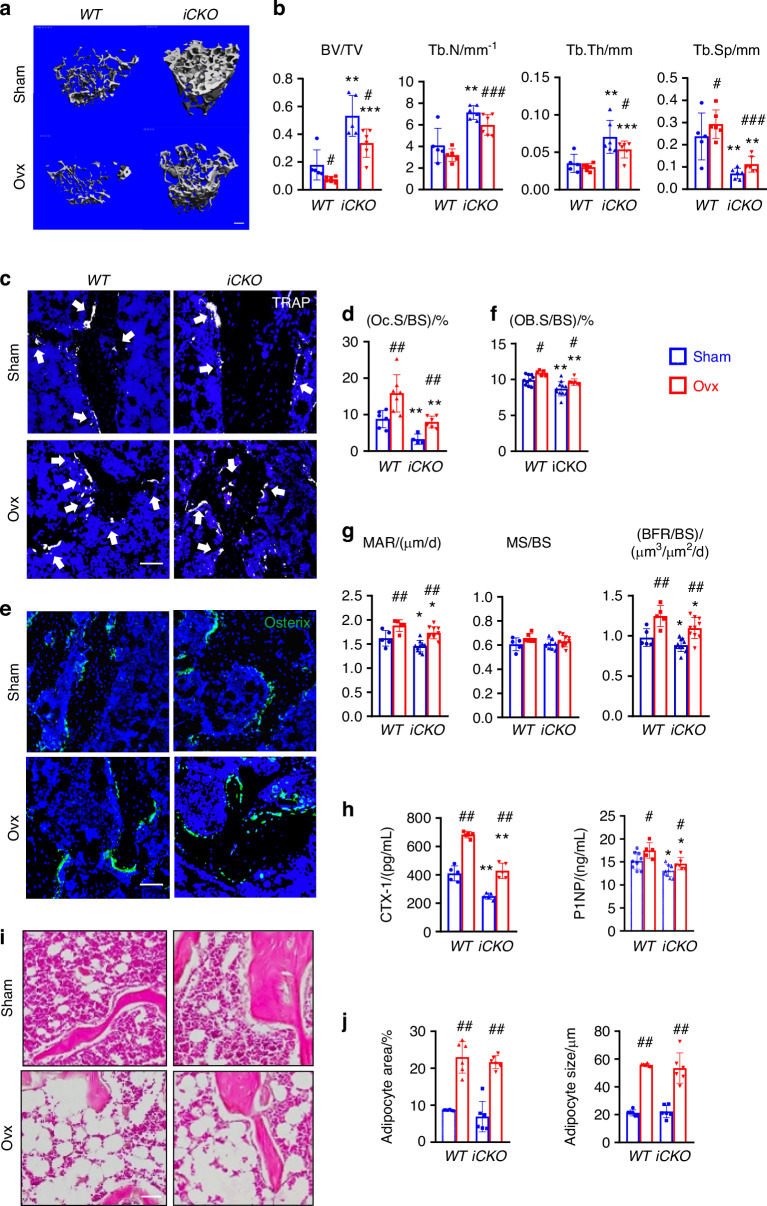
RANKL deficiency in MALPs protects adult female mice from ovariectomy-induced trabecular bone loss. **a** 3D microCT reconstruction of femoral trabecular bone from *WT* and *RANKL iCKO* mice at 6 weeks post OVX surgery. Mice received Tam injections at 3 months of age right before the surgery. Scale bar = 200 µm. **b** MicroCT measurement of trabecular bone structural parameters. **c** Representative TRAP staining images of trabecular bone from *WT* and *RANKL iCKO* femurs show TRAP^+^ osteoclasts (arrows). Scale bar = 50 μm. **d** Quantification of osteoclast surface (Oc.S). **e** Representative Osterix staining of trabecular bone from *WT* and *RANKL iCKO* femurs. Scale bar = 50 μm. **f** Quantification of osteoblast surface (OB.S). **g** Bone formation activity is quantified. **h** Serum ELISA analysis of bone resorption marker (CTX-1) and formation marker (P1NP) in *WT* and *iCKO* mice. **i** Representative H&E staining of trabecular bone from *WT* and *RANKL iCKO* femurs. Scale bar = 50 μm. **j** Quantification of the percentage of adipocyte area within bone marrow and adipocyte size. #*P* < 0.05; ##*P* < 0.01; ###*P* < 0.001 OVX vs Sham; *P < 0.05; **P < 0.01; ***P < 0.001 *iCKO* vs *WT*, *n* = 5–6 mice/group

OVX surgery in mice mimics human postmenopausal osteoporosis. To understand the functional role of MALP-derived RANKL in pathological bone loss, we injected Tam into 3-month-old female *WT* and *iCKO* mice for 3 days and subjected them to sham or OVX surgery one day after the last injection. Mice were euthanized 6 weeks later. Estrogen deficiency was confirmed by an 86.7% decrease in uterine weight of *WT* (Fig. S11). Similar changes were also observed in *iCKO* mice. In sham groups, *iCKO* mice displayed a drastically increase in femoral and vertebral trabecular bone mass (2.9-fold and 1.5-fold, respectively) compared to *WT* mice (Fig. 5a, b, Fig. S12). OVX reduced femoral trabecular BV/TV by 57.8% in *WT* mice and 36.9% in *iCKO* mice. Compared to *WT* mice, *iCKO* mice exhibited 4.5-, 1.9- and 1.8-fold increases in BV/TV, Tb.N, and Tb.Th, respectively, and a 70.3% decrease in Tb.Sp at 6 weeks post OVX (Fig. 5a, b). This preservation of trabecular bone post OVX was more prominent in vertebrae, with 53.4% and 25.8% decreases in BV/TV in *WT* and *iCKO* mice (Fig. S12), respectively. OVX did not affect femoral cortical bones in both *WT* and *iCKO* mice (Fig. S13).

Bone histomorphometry revealed that OVX increased osteoclast surface in both *WT* and *iCKO* mice but *iCKO* mice with OVX have 49.8% less osteoclast surface compared to *WT* mice with OVX (Fig. 5c, d). OVX also increased osteoblast surface and osteoblast activity in *WT* and *iCKO* mice (Fig. 5e–g). Serum chemistry further confirmed that bone turnover is increased in both genotypes but bone resorption, marked by CTX-1, is 37.2% less in *iCKO* with OVX compared to *WT* with OVX (Fig. 5h). Taken together, the above data demonstrate that RANKL from MALPs contributes to the enhanced bone resorption in the OVX model.

OVX also induces bone marrow adiposity (Fig. 5i, j). Interestingly, while MALPs are precursors for marrow adipocytes, their number did not change after OVX (Fig. S14). Compared to *WT*, we did not observe any change in marrow adipocytes in *iCKO* mice after sham surgery. After OVX, adipocyte area and size in *iCKO* mice were increased similarly as *WT* mice (Fig. 5i, j). These data suggest that RANKL from MALPs does not participate in OVX-induced marrow adiposity.

### RANKL depletion in osteoporotic bone restores bone mass

**Fig. 6:**
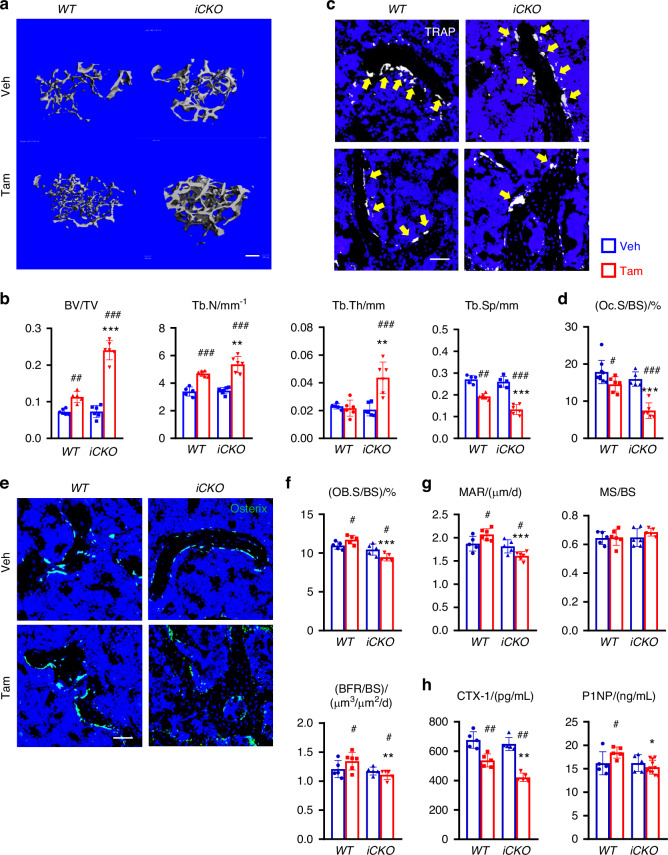
Depleting RANKL in MALPs in osteoporotic mice restores trabecular bone mass. **a** 3D microCT reconstruction of femoral trabecular bone from *WT* and *RANKL iCKO* mice at 10 weeks post OVX surgery. Mice received the surgery at 3 months of age and vehicle or Tam injections 6 weeks later. Scale bar = 200 µm. **b** MicroCT measurement of trabecular bone structural parameters. **c** Representative TRAP staining images of femoral trabecular bone from *WT* and *RANKL iCKO* mice with vehicle or Tam injections show TRAP^+^ osteoclasts (arrows). Scale bar = 50 μm. **d** Quantification of osteoclast surface (Oc.S). **e** Representative Osterix staining of femoral trabecular bone from *WT* and *RANKL iCKO* mice with vehicle or Tam injections. Scale bar = 50 μm. **f** Quantification of osteoblast surface (OB.S). **g** Bone formation activity is quantified. **h** Serum ELISA analysis of bone resorption marker (CTX-1) and formation marker (P1NP) in *WT* and *iCKO* mice with vehicle or Tam injections. #*P* < 0.05; ##*P* < 0.01; ##*P* < 0.001 Tam vs Veh; *P < 0.05; **P < 0.01; ***P < 0.001 *iCKO* vs *WT*, *n* = 5–6 mice/group

Next, we investigated whether MALPs-derived RANKL can be targeted for osteoporosis treatment. To do so, we subjected 3-month-old mice to OVX. Six weeks later when trabecular bone mass is significantly reduced, *iCKO* mice received vehicle or Tam injections for 3 days to deplete RANKL expression in MALPs. As a control, *WT* mice received similar surgery and injections. To our surprise, even 3 times of Tam injections significantly increased femoral and vertebral trabecular bone mass in *WT* mice by 1.6-fold and 1.5-fold, respectively, at 4 weeks later (Fig. 6a, b, Fig. S15), suggesting that Tam alone has beneficial effects on bone. In comparison, Tam administration increased femoral trabecular bone mass in *iCKO* mice at a much higher level (3.3-fold), accompanied by a 1.5-fold increase in Tb.N, a 2.0-fold increase in Tb.Th. and a 49.0% decrease in Tb. Sp (Fig. 6a, b). Similar effects were also observed in vertebral trabecular bone (Fig. S15).

Subsequent bone histomorphometry revealed that Tam injections in *WT* mice decrease osteoclast surface by 18.7% (Fig. 6c, d) while increasing osteoblast surface by 1.1-fold and enhancing osteoblast activity (Fig. 6e–g). Strikingly, Tam injections in *iCKO* mice greatly reduced osteoclast surfaces by 53.1% (Fig. 6c, d). Osteoblast surface was reduced by 9.6% (Fig. 6e, f) and osteoblast activity was also reduced (Fig. 6g). Serum chemistry confirmed that *iCKO* mice have a greater reduction of bone resorption than *WT* mice after Tam treatment (Fig. 6h). Taken together, our data suggest that after OVX-induced osteoporosis is established, depletion of RANKL in MALPs is still effective in restoring trabecular bone within a short period of time.

### MALP-derived RANKL contributes to bone healing after injury

**Fig. 7:**
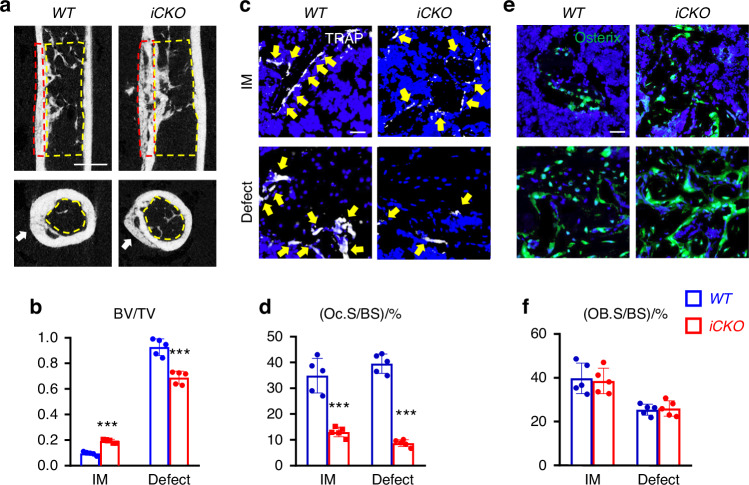
Bone healing is delayed in mice with RANKL depletion in MALPs. **a** Representative sagittal (top) and transverse (bottom) cross-sections of microCT images of drill-hole defects in *WT* and *RANKL iCKO* mice. Mice received Tam injections at 3 months of age followed by drill hole injury. Femurs were harvested at 4 weeks later for examination. Arrows point to the defect region. Yellow and red dashed squares indicate the areas for quantification of intramedullary and cortical defect regions, respectively. Scale bar = 1 mm. **b** Quantification of bone volume fraction at intramedullary (IM) and cortical defect regions. **c** Representative TRAP staining images of bone at intramedullary and cortical defect regions from *WT* and *RANKL iCKO* mice to show TRAP^+^ osteoclasts (arrows). Scale bar = 20 μm. **d** Quantification of osteoclast surface (Oc.S). **e** Representative Osterix staining of bone at intramedullary and cortical defect regions from *WT* and *RANKL iCKO* mice. Scale bar = 20 μm. **f** Quantification of osteoblast surface (OB.S). ***P < 0.001 vs *WT*, *n* = 5 mice/group

Osteoclasts play an important role in the cartilage and bone remodeling stages of fracture healing.^2^ However, whether they are also required for healing after bone defect injury is not well studied. Since Adipoq^+^ cells are located inside the bone, not at the periosteal bone surface, we next drilled non-critical size holes in the femoral cortex of *iCKO* and *WT* mice. In this injury model, trabecular bone appears first in the bone marrow close to the cortical defect region and then is resolved after healing, indicating a bone remodeling process. Meanwhile, the defect area is filled with new bone via intramembranous ossification. We carried out the drill-hole injury on mice 4 days after daily Tam injections at day 1-3. MicroCT analysis showed that the hole in *WT* mice is healed nicely at 4 weeks post injury with almost no intramedular trabecular bone left (Fig. 7a, b). However, *iCKO* mice still had a significant amount of trabecular bone remaining. Compared to *WT* mice, *iCKO* mice showed decreased BV/TV in the cortical bone area (25.7%) and increased BV/TV in the intramedullary area (2.0-fold), indicating a delayed healing. Histomorphometry analysis showed that osteoclasts in *iCKO* mice are drastically reduced by 62.7% in the defect cortical bone area and 78.0% in the intramedular trabecular bone area (Fig. 7c, d), while osteoblasts are not affected (Fig. 7e, f). Our data indicate that MALP-derived RANKL drives osteoclastogenesis and bone remodeling in this type of bone repair.

## Discussion

Our prior studies, as well as others, revealed that MALPs-derived osteoclast regulatory cytokines, RANKL and Csf1, are important for trabecular bone remodeling in young mice.^19–22^ However, those studies used constitutive *Adipoq-Cre* and thus did not address their actions in adult bone tissue. In this report, we first analyzed mouse and human scRNA-seq datasets and utilized in situ experiments to discover that RANKL and Csf1 are mainly expressed in MALPs but not in osteoblasts and osteocytes. We then studied adult bone phenotypes of RANKL deficient mice at two anatomic sites (long bone and vertebra) using inducible *Adipoq-CreER* under normal and pathological conditions. Collectively, our data demonstrate that RANKL derived from marrow adipogenic precursors plays a dominant role in stimulating osteoclast formation and promoting trabecular bone resorption under normal and pathological conditions. This conclusion is consistent with a previous report revealing that adipogenic transcription factors C/EBPβ and δ promotes RANKL expression in mesenchymal progenitors undergoing adipogenic differentiation.^27^

Prior studies using osteocyte-specific *Cre*s, such as *Dmp1-Cre* and *Sost-Cre*, to ablate RANKL proposed that bone embedding osteocytes are crucial for trabecular bone remodeling.^12–14^ Our research challenges this conventional view. First, scRNA-seq of bone marrow mesenchymal lineage cells in mouse and human samples revealed a specific expression of RANKL in MALPs. Second, in situ staining of *Rankl* clearly showed that *Rankl* is mainly expressed in cells expressing *Adipoq* and *Pparg*, two markers for MALPs. To our surprise, we did not detect *Rankl* mRNA in osteocytes, which might reflect a relatively low sensitivity of in situ approach. A prior report detected RANKL expression in one third of osteocytes using RANKL antibody.^28^ However, their immunohistology images showed more RANKL^+^ cells in the bone marrow. Third, *RANKL iCKO* mice exhibited a striking 3.3-fold increase of femoral trabecular bone mass within one month of RANKL depletion, which is higher than ~2.5-fold increase in 6-month-old *Dmp1-Cre RANKL CKO* mice.^13^ At this time point, only 9.9% of osteocytes and 46.1% of osteoblasts in the trabecular bone are labeled by Td in *AdipoqER/Td* mice. Fourth, *Dmp1-Cre* is not specific for osteocytes. Lineage tracing revealed that it also labels all osteoblasts and ~30% CAR cells,^29^ a mesenchymal subpopulation highly overlapped with MALPs. These data are in line with the low *Dmp1* expression in LCP and osteoblast clusters in our scRNA-seq.^17^ Thus, it is likely that *Dmp1-Cre* driven RANKL knockout depletes RANKL in MALPs as well. *Sost-Cre* is more specific for osteocytes. However, it also labels many hematopoietic cells.^12^ Some of them, such as B and T lymphocytes, express RANKL too.^30–32^ Lastly, our proposal that MALPs are a predominant source of RANKL in the trabecular bone also fits well with the emerging view that osteoclasts are long-lived cells constantly undergoing recycling.^3^ Using intravital microscope, McDonald et al. discovered that RANKL rapidly stimulates fusion and fission of osteomorphs, small daughter cells of osteoclasts.^6^ Since osteomorphs reside only in the bone marrow, MALPs in the bone marrow but not osteocytes in the bone matrix are more likely to be the major RANKL source regulating their activities. That being said, we cannot exclude the importance of osteocytes-derived RANKL in diseases and treatment conditions not investigated in this research. Due to their abundance in the cortical bone, osteocytes are likely to be the major regulator of cortical bone turnover. This is supported by findings from Xiong et al., which demonstrated that mice with *Dmp1-Cre*-driven RANKL knockout are resistant to tail suspension–induced cortical bone loss.^13^

In addition to osteoporosis, osteoclasts are also important for bone healing. Past research in this field focused on fracture, which is mostly repaired via an endochondral ossification mechanism.^33^ Previous studies showed that suppressed bone resorption, either by RANK depletion^34^ or by pharmacological inhibition of RANKL,^35^ delays cartilage dissolution and callus remodeling and thus reduces bony unions. On the contrary, increased RANKL activity by depleting OPG, the decoy receptor for RANK, stimulates osteoclastogenesis and accelerates bone fracture healing.^36^ To our knowledge, this study is the first investigation of osteoclasts in bone healing after drill hole injury, which is repaired via an intramembranous ossification mechanism. It is interesting to note that RANKL depletion in MALPs does not affect cortical bone during bone maintenance and after estrogen deficiency but delays cortical bone healing after drill hole injury. In *RANKL iCKO* mice, reduced osteoclastogenesis at the injury site causes persistent remaining of bony callus, leading to delayed healing at the injury site.

Bone surface is covered by osteoblasts, osteoclasts, and bone lining cells. Compared to osteoblasts with a large, cuboidal shape, bone lining cells are morphologically defined as flattened cells covering quiescent bone surface not undergoing bone remodeling. In the conventional view, they are descendants of osteoblasts and able to be quickly re-activated into osteoblasts upon stimulations, such as PTH, mechanical loading, and radiation.^37–39^ A prior study also found that they can be a major source of osteoblasts during adulthood.^40^ However, it is puzzling that scRNA-seq analyses performed so far have not identified a subpopulation matching the above characteristics of bone lining cells. To our surprise, we found many Td^+^ cells on the trabecular and endocortical bone surface in adult *AdipoqER/Td* mice. Those Adipoq^+^ bone surface cells are Osterix- cells, and hence not osteoblasts. Some of them express *Pparg*, *Rankl*, or *Csf1* mRNAs, which are highly specific for MALPs based on scRNA-seq. These data suggest that bone lining cells may contain adipogenic cells. Future research using spatial omics techniques will define the composition of bone lining cells and provide new insights into bone remodeling.

One limitation of our study is that *Adipoq-CreER* does not solely label MALPs. In our study, *Adipoq-CreER* instantly marks adipocytes and *Pparg*-expressing cells while gradually marking osteoblasts and osteocytes over time. This indicate that in addition to MALPs, it also labels mesenchymal progenitors capable of bilineage differentiation. The same labeling pattern has been reported by other researchers.^23,24^ Since Adipoq^+^ cells do not form CFU-Fs and do not expand over time, those additional bipotent progenitors are likely to be late-stage mesenchymal progenitors, such as LCPs identified in our scRNA-seq. This leads to a possible depletion of RANKL in osteogenic cells in our mouse model. Nevertheless, since our studies focus on 2-6 weeks after Tam injection, a time point when the majority of osteocytes are not labeled by Td, we believe our conclusion that MALP-derived RANKL plays a dominant role in trabecular bone resorption is still valid.

In conclusion, we have demonstrated that bone marrow adipoprogenitors control bone resorption at the trabecular bone region in adult mice during homeostasis and pathological conditions. Currently, Denosumab, an FDA-approved human monoclonal antibody to RANKL, is the first line osteoporosis drug.^41^ It also shows great promise in other diseases, such as bone tumors and autoimmune diseases. Our research discovering that MALPs are the major source of RANKL in adult bone marrow sheds new insight into targeting this important signaling pathway in various diseases. With the advance in drug design and delivery, future studies should focus on how to manipulate this cell population to achieve the best clinical efficacy with minimum side effects.

## Material and methods

### Analysis of scRNA-seq datasets

Pre-aligned scRNA-seq matrix files were acquired from GEO GSE145477 and GSE176171 (mouse) and GSE253355 (human). Standard Seurat pipeline^42^ was used for filtering, normalization, variable gene selection, dimensionality reduction analysis and clustering. For the integrated dataset, batch integration was performed using Harmony (version 1.0).^43^ Cell type was annotated according to the metadata from published datasets.^15,26^

### Animals study design

All animal work performed in this report was approved by the Institutional Animal Care and Use Committee (IACUC) at the University of Pennsylvania. *Adipoq-CreER Rosa-tdTomato* (*AdipoqER/Td*) mice were generated by breeding *Rosa-tdTomato*^44^ mice with *Adipoq-CreER* mice.^45^ To generate *RANKL iCKO* mice, we first bred *Adipoq-CreER* with *RANKL*^*flox/flox*^ mice^20^ to obtain *Adipoq-CreER RANKL*^*flox/+*^, which were then crossed with *RANKL*^*flox/flox*^ to generate *RANKL iCKO* mice. Male *RANKL iCKO* mice was further crossed with female *RANKL*^*flox/flox*^ mice to generate *RANKL iCKO* mice and *WT* (*RANKL*^*flox/flox*^) siblings. All mouse lines, except *RANKL*^*flox/flox*^, were obtained from Jackson Laboratory (Bar Harbor, ME, USA). To induce Td expression and RANKL depletion, mice at 3 months of age received daily intraperitoneal injections of Tam (75 mg/kg) for 3 days. For OVX surgery, 3-month-old female mice received either OVX or sham operation and their femurs, tibiae, and vertebrae were collected 6 or 10 weeks later for analyses. For drill hole injury, 3-month-old female mice received a 0.8-mm diameter unicortical drill hole defect via a 21 G needle at the diaphysis part of right femurs and their injured femurs were collected 4 weeks later for analyses.

### Micro-computed tomography (microCT) analysis

MicroCT analysis (microCT 45, Scanco Medical AG, Brüttisellen, Switzerland) was performed at 7.4 µm isotropic voxel size as described previously.^46^ Briefly, the distal end of femur corresponding to a region at 0–3.4 mm below the growth plate was scanned. The images of the secondary spongiosa regions (0.6–2.1 mm below the lowest point of the growth plate (GP), ~200 slices) were contoured for trabecular bone analysis. At the femur midshaft, 100 slices located at 4.7–5.5 mm away from the distal growth plate were acquired for cortical bone analyses. In vertebrae, the region 50 slices away from the top and bottom end plates (~300 slices) was acquired for trabecular bone analysis. To analyze bone healing after drilling a hole, the contouring of defect area or intramedullary area were manually defined. A total of 150 slices were used for trabecular bone analysis. Trabecular and cortical bones were segmented from soft tissue using a threshold of 487.0 mgHA/cm^3^ and 661.6 mgHA/cm^3^, respectively, with a Gaussian noise filter (sigma = 1.2, support = 2.0). For trabecular bone analysis, trabecular bone volume fraction (BV/TV), trabecular thickness (Tb.Th), trabecular separation (Tb.Sp), and trabecular number (Tb.N) were recorded. For cortical bone analysis, periosteal perimeter (Ps.Pm), endosteal perimeter (Ec.Pm), cortical bone area (Ct.Ar), cortical thickness (Ct.Th), and tissue mineral density (TMD) were recorded. All calculations were performed based on 3D standard microstructural analysis.^47^

### Human sample collection

Femoral heads were de-identified surgical discard specimens obtained from 3 patients undergoing total hip arthroplasty surgery. Morphologically normal bone marrow tissues were sampled at the distal site to the articular cartilage for histology analysis.

### Histology

To obtain cryosections without decalcification, mouse bones were dissected and fixed in 4% paraformaldehyde (PFA) for 24 h, dehydrated in 30% sucrose, embedded in optimal cutting temperature (OCT) compound, and sectioned at 6 μm in thickness using cryofilm tape (Section Lab, Hiroshima, Japan). For immunostaining, sections were incubated with rabbit anti-Osterix (Abcam, ab22552), rat anti-CD45 (Biolegend, 103101), rat anti-Endomucin (Santa Cruz, sc-65495) or rabbit anti-Perilipin (Cell signaling, 9349) at 4 °C overnight followed by Alexa Fluor 488 anti-rat (Abcam, ab150155) or Alexa Fluor 647 anti-rabbit (Abcam, ab150075) secondary antibodies incubation 1 h at RT. Fluorescent TRAP staining was performed as described previously.^48^ Sections were scanned by Axioscan (Carl Zeiss MicroImaging, Göttingen, Germany). In the lineage tracing experiment, we selected the following areas in distal femurs to count Td^+^ bone marrow cells: subchondral bone, top metaphysis (0.6–2.1 mm distal to GP), bottom metaphysis (3.1–4.6 mm distal to GP), and diaphysis (6.5–8.0 mm distal to GP).

To measure dynamic histomorphometry, mice received calcein (10 mg/kg, Sigma Aldrich) and xylenol orange (90 mg/kg, Sigma Aldrich) at 9 and 2 days, respectively, before euthanization. Areas within the secondary spongiosa of tibiae were quantified by OsteoMeasure Software (OsterMetrics, Decatur, GA, USA). The primary indices include total tissue area (TV), trabecular bone perimeter (BS), single- and double-labeled surface (s/dLS), and interlabel width. Mineralizing surface (MS), bone formation rate (BFR), and surface-referent bone formation rate (BFR/BS, μm^3^/μm^2^/d) were calculated as described by Dempster et al.^49^

To obtain paraffin sections, femurs were fixed in 4% PFA for 24 h and decalcified in a 10% EDTA for 4 weeks at 4 °C. Samples were then embedded in paraffin, sectioned at 6 μm in thickness, and processed for H&E staining and Safranin O/fast green staining.

After collection, human bone samples were fixed in 4% PFA overnight, decalcified by Morse solution (10% sodium citrate and 22.5% formic acid) for 3 days, after rinsing the samples three times with RNase-Free water, proceed with paraffin embedding for sectioning.

For RNA FISH experiment, we adopted in situ hybridization chain reaction (HCR) approach (Molecular Instruments, Los Angeles, CA). For mouse samples, cryosections were processed and stained by probes against *Rankl* (NM_011613.4), *Csf1* (NM_001113529.1), and *Pparg* (NM_001127330.3) mRNAs according to manufacturer’s protocol (HCR™ RNA-FISH protocol for fresh frozen or fixed frozen tissue sections). For human samples, paraffin sections were stained by Human probes against *RANKL* (NM_003701.4)*, PPARG* (NM_001330615.4) mRNAs.

### Hematopoietic phenotyping

Bone marrow was flushed from mouse femurs and pre-treated with Fc-blocker (Invitrogen, 14-0161-81). After washing, bone marrow cells were stained with CD45 AF700 (Biolegend, 103205), CD170 FITC (Biolegend, 155503), Ly6G APC (Biolegend, 127605), CD115 PE-CY7 (Biolegend, 135523), Ly6C Percp (Biolegend, 128027), and CD11b BV605 (Biolegend, 563015). Peripheral blood cells were collected from mouse tail vein, processed for red blood cell lysis using PharmLyse (BD Pharmingen, 555899). To analyze T cells and B cells, peripheral blood cells were stained with CD45 AF700 (Biolegend, 103205), CD11b BV605 (Biolegend, 563015), CD3 FITC (Biolegend, 100203) and B220 Percp (Biolegend, 103233). To analyze myeloid lineage, cells were stained with CD45 AF700 (Biolegend, 103205), CD170 FITC (Biolegend, 155503), Ly6G APC (Biolegend, 127605), CD115 PE-CY7 (Biolegend, 135523), Ly6C Percp (Biolegend, 128027), and CD11b BV605 (Biolegend, 563015). Flow cytometry experiments were performed by BD LSRFortessa flow cytometer and analyzed by FlowJo v10.5.3 for WIN.

### Colony-forming unit fibroblast (CFU-F) assay

Bone marrow cells were flushed from mouse long bones and seeded at 3 × 10^6^ cells per T25 flask in growth medium (α-MEM supplemented with 15% FBS, 0.1% β-mercaptoethanol, 20 mmol/L glutamine, 100 IU/mL penicillin, and 100 µg/mL streptomycin) for 7 days before counting CFU-F number under the fluorescence inverted microscope (Leica, Germany) using bright field and fluorescence channel.

### ELISA assays

Sera were collected during mouse euthanization for measuring bone turnover markers, collagen type I C-telopeptide degradation products (mouse CTX-I ELISA Kit, MyBioSource), N-terminal propeptide of type I procollagen (Immunotag™ Mouse PINP ELISA Kit, G-Bioscience) according to the manufacturer’s instructions. Bone marrow collected by centrifugation was used to measure RANKL amount (PeproTech, 900-K233K).

### qRT-PCR analysis

Bone marrow was centrifuged from long bones and mixed with Tri Reagent (Sigma Aldrich) for RNA purification. Cortical bone was dissected from the remaining marrow-free bones, crushed in liquid nitrogen, mixed and homogenized with Tri Reagent on ice (Sigma Aldrich) for RNA purification. A Taqman Reverse Transcription Kit (Applied BioSystems, Inc., Foster City, CA, USA) was used to reverse transcribe mRNA into cDNA. The power SYBR Green PCR Master Mix Kit (Applied BioSystems, Inc) was used for quantitative real-time PCR (qRT-PCR). Primers for *Tnfsf11* gene are 5’- GGAAGCGTACCTACAGACTA-3’ (forward) and 5’- TGCTCCCTCCTTTCATCA-3’ (reverse), and primers for β-actin gene are 5’- TCCTCCTGAGCGCAAGTACTCT-3’(forward) and 5’-CGGACTCATCGTACTCCTGCTT-3’ (reverse).

### Statistical analyses

Data are expressed as means ± standard deviation (SD). For comparisons between two groups, unpaired two-sample Student’s *t* test was applied. For comparisons amongst multiple groups across two fixed effect factors (e.g., genotype and surgery), two-way ANOVA was applied, followed by Tukey-Kramer multiple comparison test to account for family-wise type I error using Prism 8 software (GraphPad Software). Values of *P* < 0.05 were considered statistically significant.

## Supplementary information

Supplementary Materials

## Data Availability

All the data support the figures and the other findings are available upon reasonable request to the corresponding author.
