# Supplementary material for: Bone marrow adipogenic lineage precursors are the major regulator of bone resorption in adult mice
Source: Bone Res. 2025 Mar 19;13:39. doi: 10.1038/s41413-025-00405-4 (PMC11920254; doi:10.1038/s41413-025-00405-4)

Figure legend

Figure S1. Bone marrow mesenchymal subpopulations in mouse scRNA-seq dataset.

(A) Heatmap of marker genes in mesenchymal subpopulations.

(B) Dot plot of MALP marker genes split by mouse age (1- and 16-month-old). EMP: early mesenchymal progenitor; LMP: late mesenchymal progenitor; LCP: lineage committed progenitor; OB: osteoblast; Ocy: osteocyte; MALP: marrow adipogenic lineage precursor.

Figure S2. Dot plot shows the expression of *Tnfsf11* and *Csf1* in mouse bone marrow mesenchymal subpopulations.

Figure S3. Dot plot shows the expression of *TNFSF11* and *CSF1* in human bone marrow cells. VSMC: vascular smooth muscle cell; SEC: sinusoidal endothelial cell; AEC: arterial endothelial cell; CLP: common lymphoid progenitor; pDC: plasmacytoid dendritic cell; Cycling DCs: cycling dendritic cells; Ba/Eo/Ma: basophil/eosinophil/mast cell; GMP: granulocyte-macrophage progenitor; RBC: red blood cell; MEP: megakaryocyte-erythroid progenitor; Cycling HSPC: cycling hematopoietic stem and progenitor cells; MPP: multipotent progenitor; HSC: hematopoietic stem cell.

Figure S4. RNA FISH reveals costaining of RANKL and PPARG mRNA in human bone marrow. Arrows point to a few double positive cells on trabecular bone surface. Scale bar=20 μm.

Figure S5. *RANKL iCKO* mice have normal trabecular and cortical bone structure without Tam

induction.

(A) 3D microCT reconstruction of femoral secondary spongiosa region from *WT* and *RANKL iCKO* mice at 3 months of age. Scale bar= 200 μm.

(B) MicroCT measurement of trabecular bone structural parameters. BV/TV: bone volume fraction; Tb.N: trabecular number; Tb.Th: trabecular thickness; Tb.Sp: trabecular separation.

(C) 3D microCT reconstruction of vertebral trabecular bone. Scale bar= 200 μm.

(D) MicroCT measurement of trabecular bone structural parameters in L4 vertebrae.

(E) 3D microCT reconstructions of femoral midshaft region from *WT* and *RANKL iCKO* mice at 3 months of age. Scale bar=200 μm.

(F) MicroCT measurement of cortical bone structural parameters in femurs. Ps.Pm: periosteal perimeter; Ec.Pm: endosteal perimeter; Ct.TMD: cortical tissue mineral density. Ct.Ar: cortical area; Ct.Th: cortical thickness.

n=5-8 mice/group.

Figure S6. *RANKL iCKO* mice are grossly normal after Tam treatment.

(A) Body weight of *WT* and *RANKL iCKO* mice was measured at 4 weeks after Tam injection. Mice received Tam at 3 months of age. n=5 mice/group.

(B) Representative Safranin O/fast green staining of long bone sections. Scale bar=250 μm.

(C) Quantification of femoral growth plate thickness. n=6 mice/group.

(D) Quantification of tibial length. n=6 mice/group.

Figure S7. RANKL depletion in MALPs rapidly increases long bone trabecular bone mass in adult mice.

(A) 3D microCT reconstruction of femoral trabecular from *WT* and *iCKO* mice at 2 weeks after Tam injections. Mice received Tam at 3 months of age. Scale bar=200 µm.

(B) MicroCT measurement of trabecular bone structural parameters.

(C) Representative TRAP staining images show TRAP+ osteoclasts (arrows) at different skeletal sites: secondary spongiosa (SS), chondro-osseous junction (COJ), and endosteal surface (Endo.S). Scale bar=50 µm.

(D) Quantification of osteoclast surface (Oc.S) at 3 skeletal sites. BS: bone surface. L: COJ length.

(E) Representative Osterix staining of trabecular bone from *WT* and *RANKL iCKO* femurs. Scale bar=50 µm.

(F) Quantification of osteoblast surface (OB.S).

*: $p<0.05$; **: $p<0.01$; ***: $p<0.001$ vs *WT*, n=5-6 mice/group.

Figure S8. RANKL deficiency in MALPs does not affect cortical bone structure.

(A) 3D microCT reconstruction of femoral midshaft region from *WT* and *iCKO* mice at 2 and 4 weeks after Tam injection. Mice received Tam at 3 months of age. Scale bar=200 µm.

(B) MicroCT measurement of cortical bone structural parameters. n=5-6 mice/group.

Figure S9. Depletion of RANKL in MALPs increases vertebral trabecular bone mass in adult mice.

(A) 2D microCT reconstruction of vertebrae from *WT* and *iCKO* mice at 2 and 4 weeks after Tam injections. Mice received Tam at 3 months of age. Scale bar=200 µm.

(B) MicroCT measurement of trabecular bone structural parameters in vertebrae. *, $p<0.05$; **, $p<0.01$; ***, $p<0.001$, n=5–6 mice/group.

Figure S10. RANKL deficiency in MALPs does not affect blood cell production.

(A) Flow analysis of hematopoietic cells in bone marrow of *WT* and *RANKL iCKO* mice at 1 month post Tam injections.

(B) Flow analysis of peripheral blood from these mice.

(C) Spleen weight of these mice.

n=6–7 mice/group.

Figure S11. Examination of mouse uterine weight after ovariectomy surgery.

Uterine weight of *WT* and *RANKL iCKO* mice at 6 weeks after OVX. Mice at 3 months of age received Tam injections followed by sham or OVX surgery. ###: $p<0.001$, OVX vs Sham. n=5 mice/group.

Figure S12. RANKL deficiency in MALPs protects adult female mice from ovariectomy-induced vertebral trabecular bone loss.

(A) 2D microCT reconstruction of vertebral trabecular bone from *WT* and *RANKL iCKO* mice at 6 weeks post OVX surgery. Mice received Tam injections at 3 months of age before the surgery. Scale bar: 200 μm.

(B) MicroCT measurement of trabecular bone structural parameters. #: $p<0.05$; ##: $p<0.01$; ###: $p<0.001$ OVX vs Sham; *: $p<0.05$; **: $p<0.01$; ***: $p<0.001$ *iCKO* vs *WT*, n=5-6 mice/group.

Figure S13. Ovariectomy and RANKL depletion in MALPs do not affect cortical bone structure.

(A) 3D microCT reconstruction of femoral cortical bone from *WT* and *RANKL iCKO* mice at 6 weeks post OVX surgery. Mice received Tam injections at 3 months of age before the surgery.

Scale bar=200 µm.

(B) MicroCT measurement of cortical bone structural parameters. n=5 mice/group.

Figure S14. Ovariectomy does not alter the number of MALPs in the bone marrow.

(A) Fluorescent images of bone marrow from *AdipoqER/Td* mice at 6 weeks post sham or OVX surgery. Mice at 3 months of age received Tam injections before surgery. Scale bar=50 µm.

(B) Quantification of Td+ cells in the bone marrow. n=6-7 mice/group.

Figure S15. Depleting RANKL in MALPs in osteoporotic mice restores trabecular bone mass in vertebrae.

(A) 2D microCT reconstruction of vertebral trabecular bone from *WT* and *RANKL iCKO* mice at 10 weeks post OVX surgery. Mice received OVX surgery at 3 months of age and vehicle or Tam injections 6 weeks later. Scale bar=200 µm.

(B) MicroCT measurement of vertebral trabecular bone structural parameters. ##: $p<0.01$; ###: $p<0.001$ Tam vs Veh; *: $p<0.05$; **: $p<0.01$; ***: $p<0.001$ *iCKO* vs *WT*, n=5-6 mice/group.

Figure S1

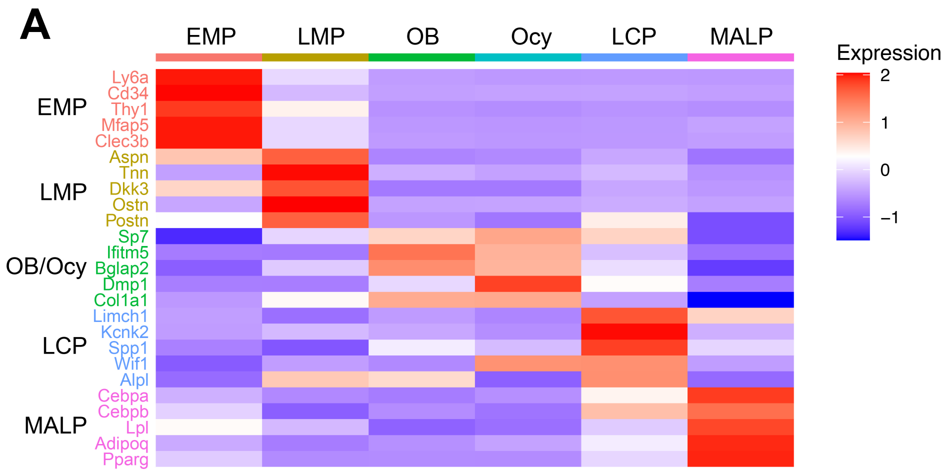

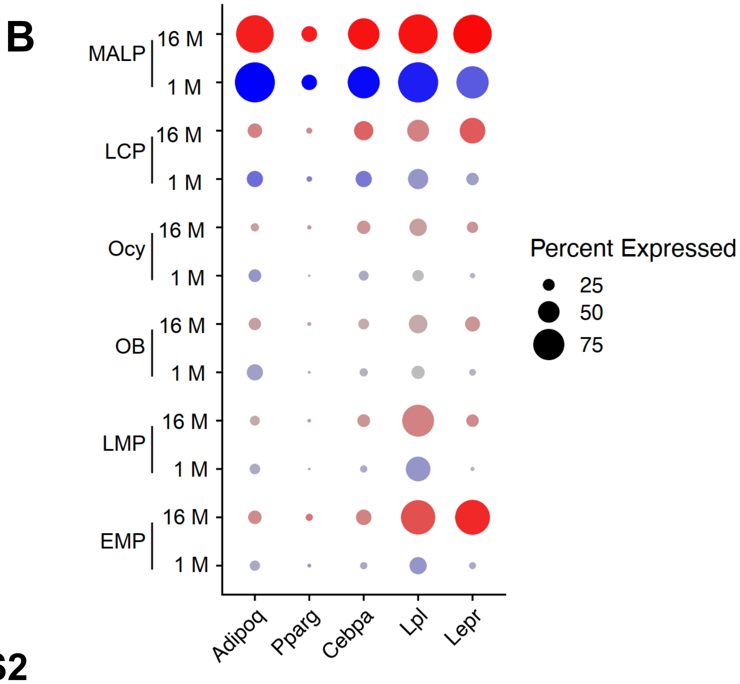

Figure S2

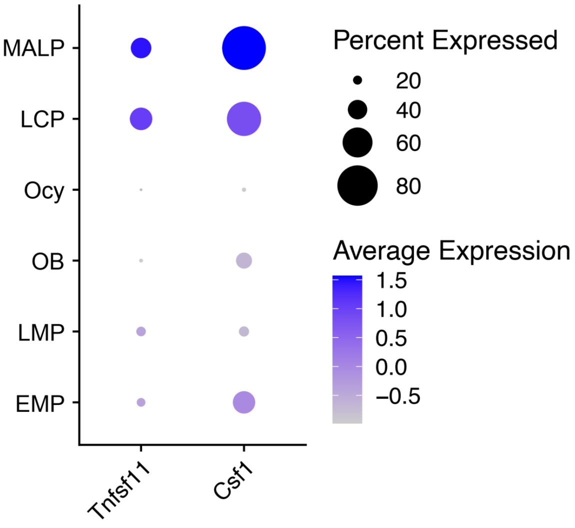

Figure S3

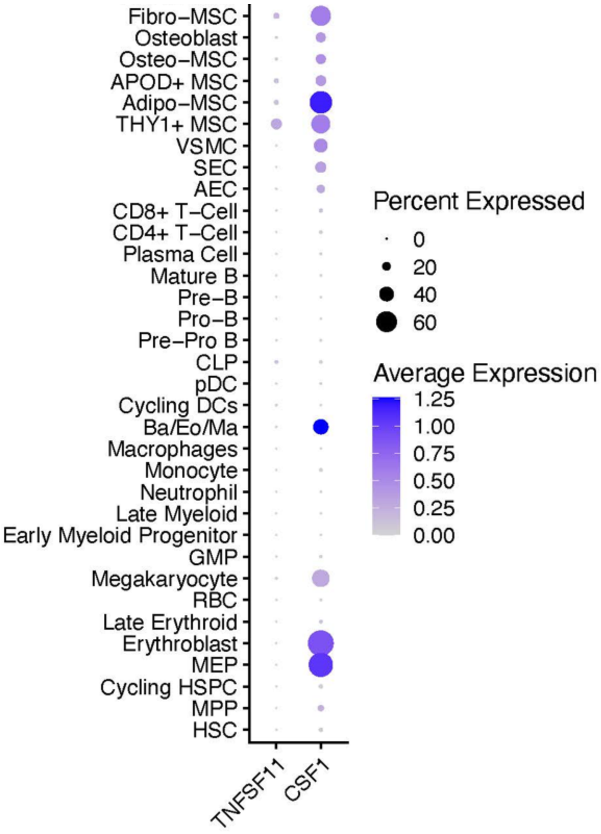

Figure S4

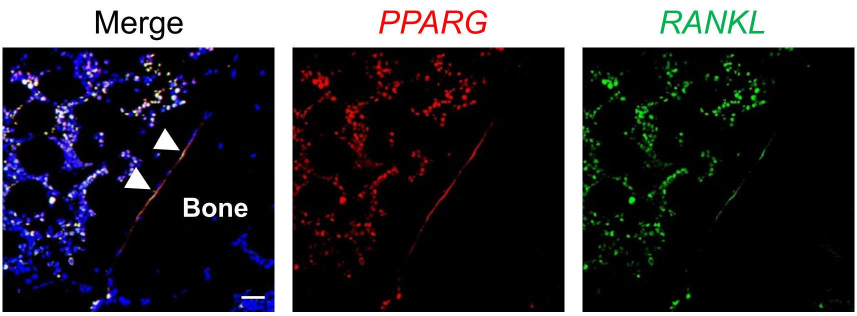

Figure S5

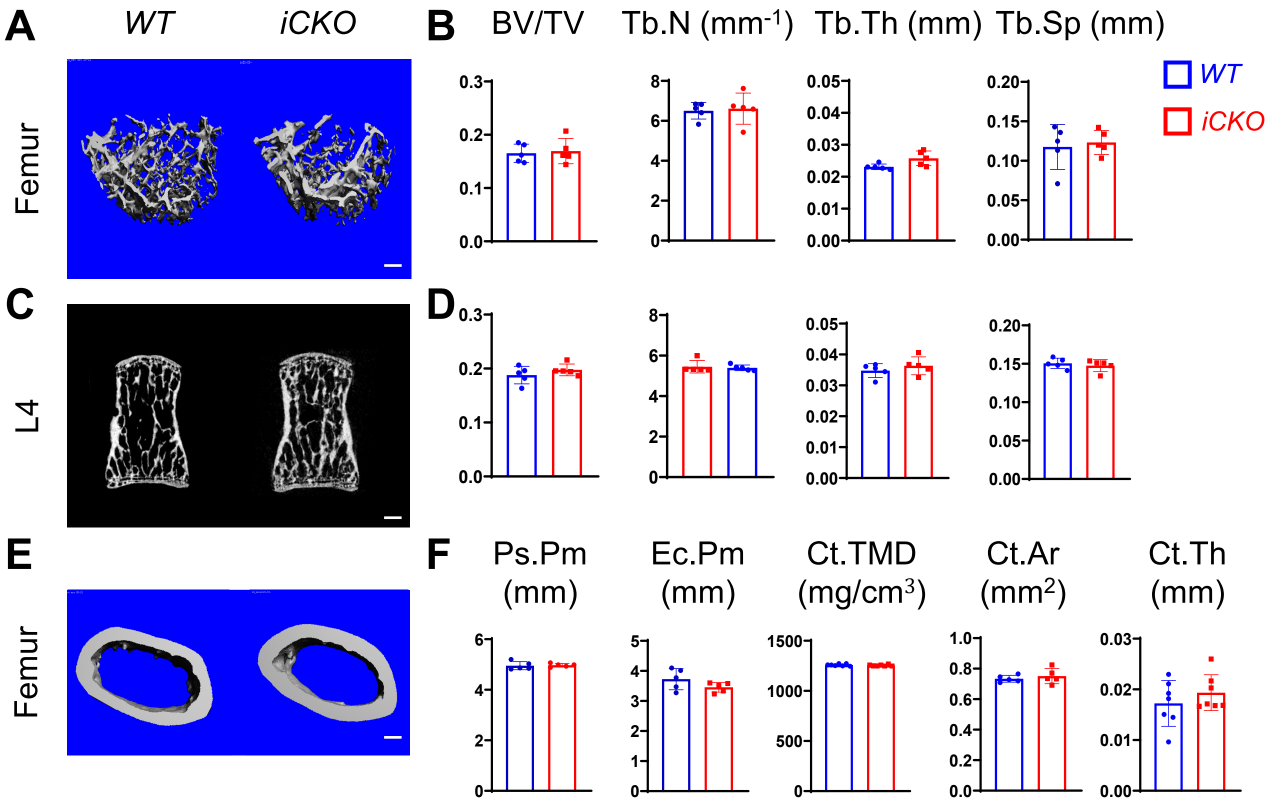

Figure S6

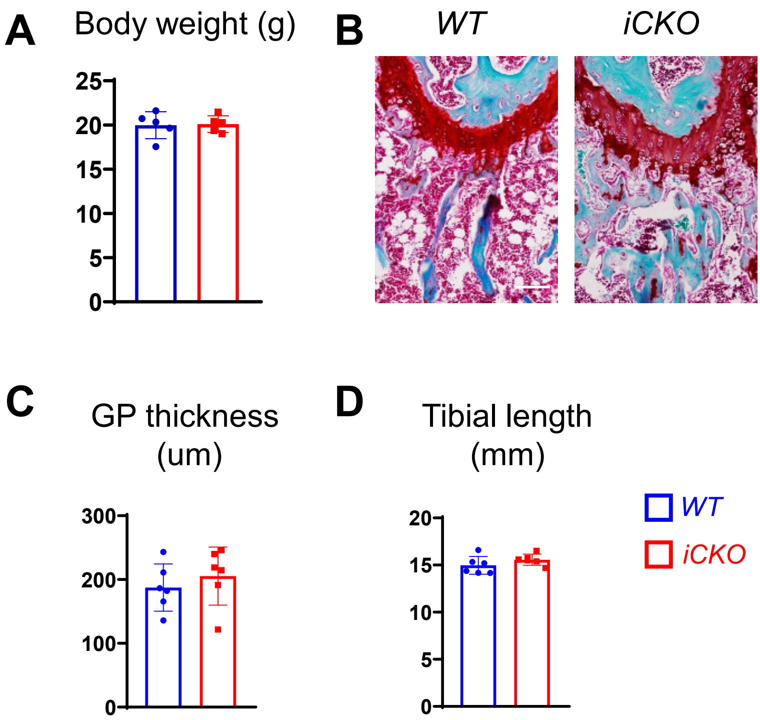

Figure S7

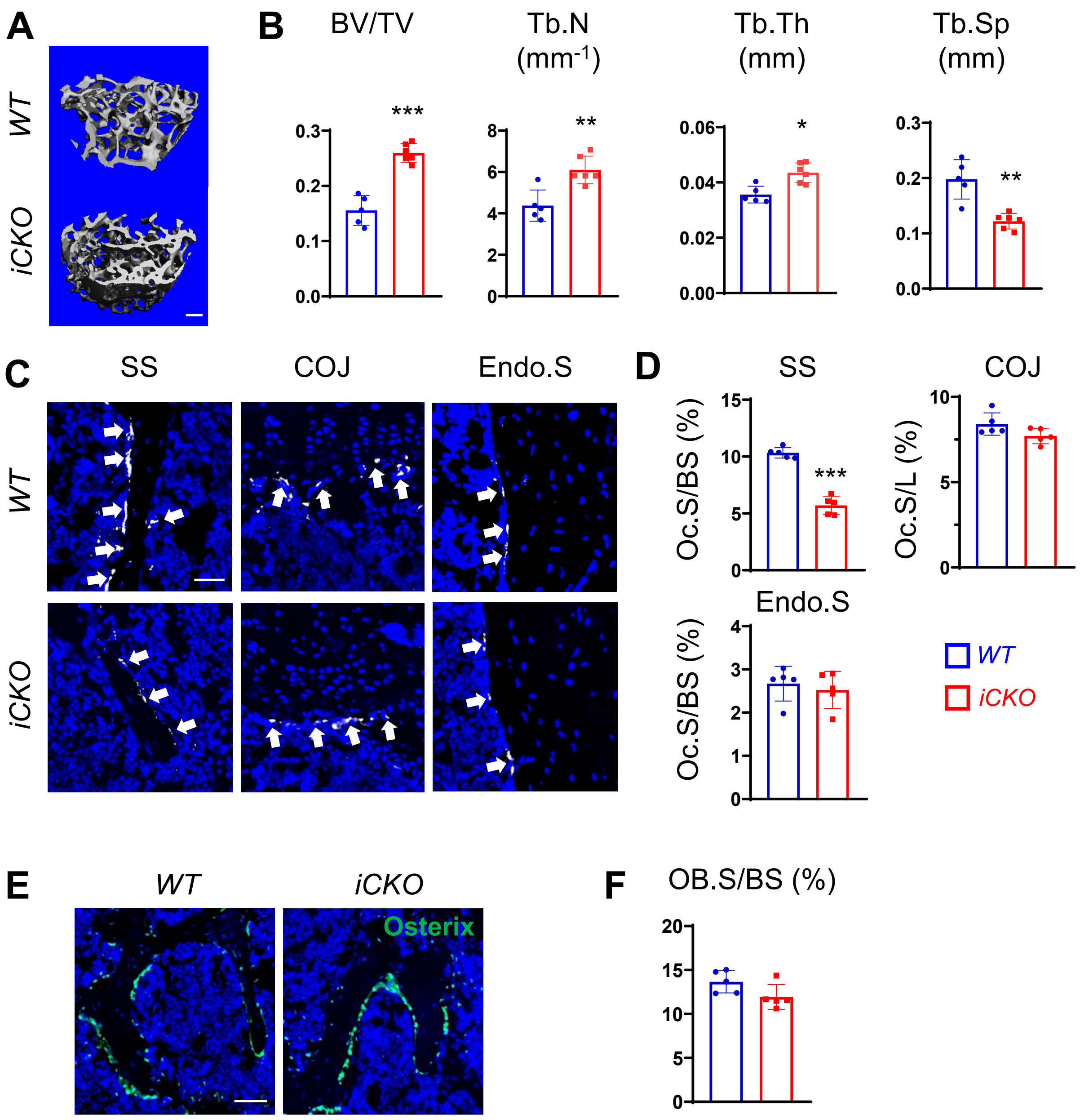
A
WT
iCKO
B
BV/TV
Tb.N (mm^{-1})
Tb.Th (mm)
Tb.Sp (mm)
C
SS
COJ
Endo.S
D
Oc.S/BS (%)
Oc.S/L (%)
WT
iCKO
E
Osterix
F
OB.S/BS (%)

Figure S8

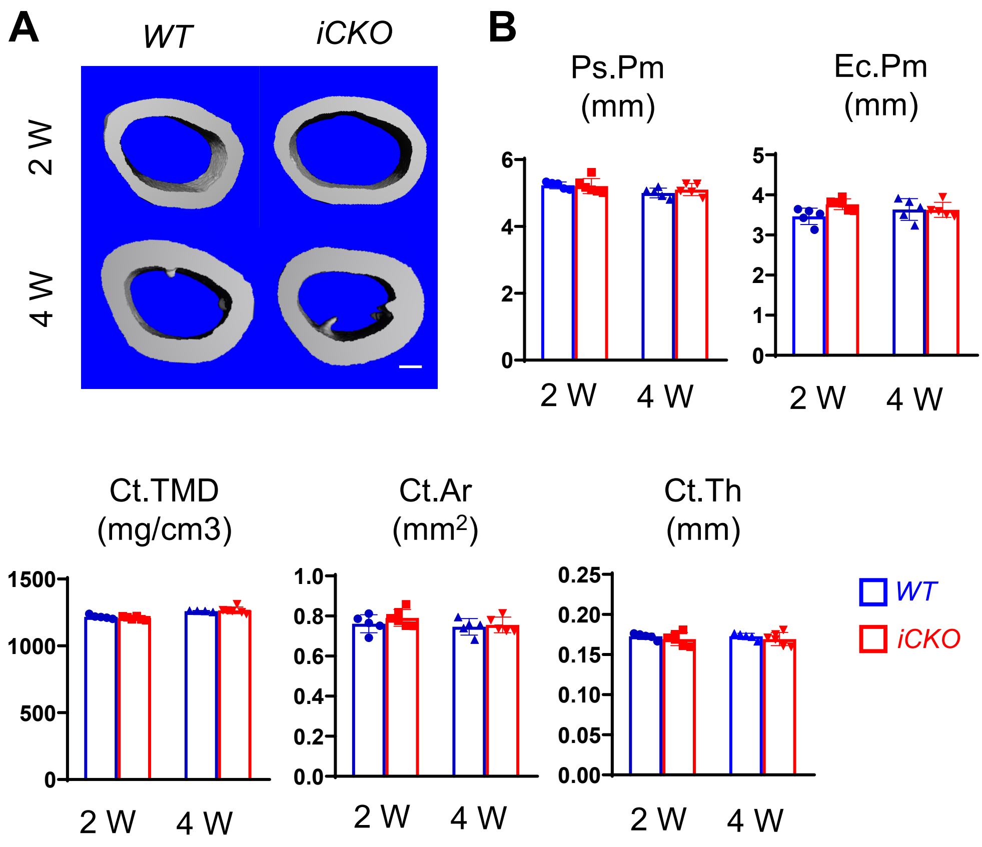

Figure S9

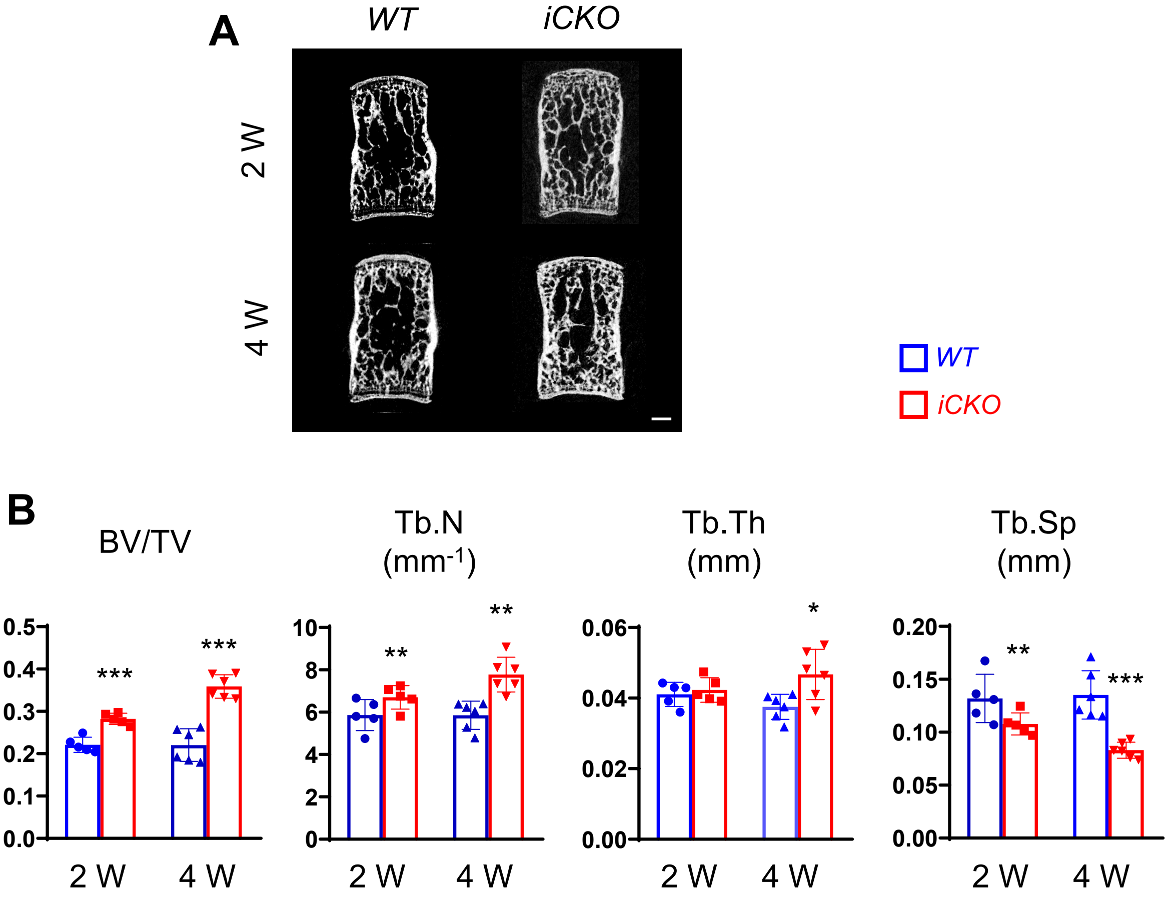

Figure S10

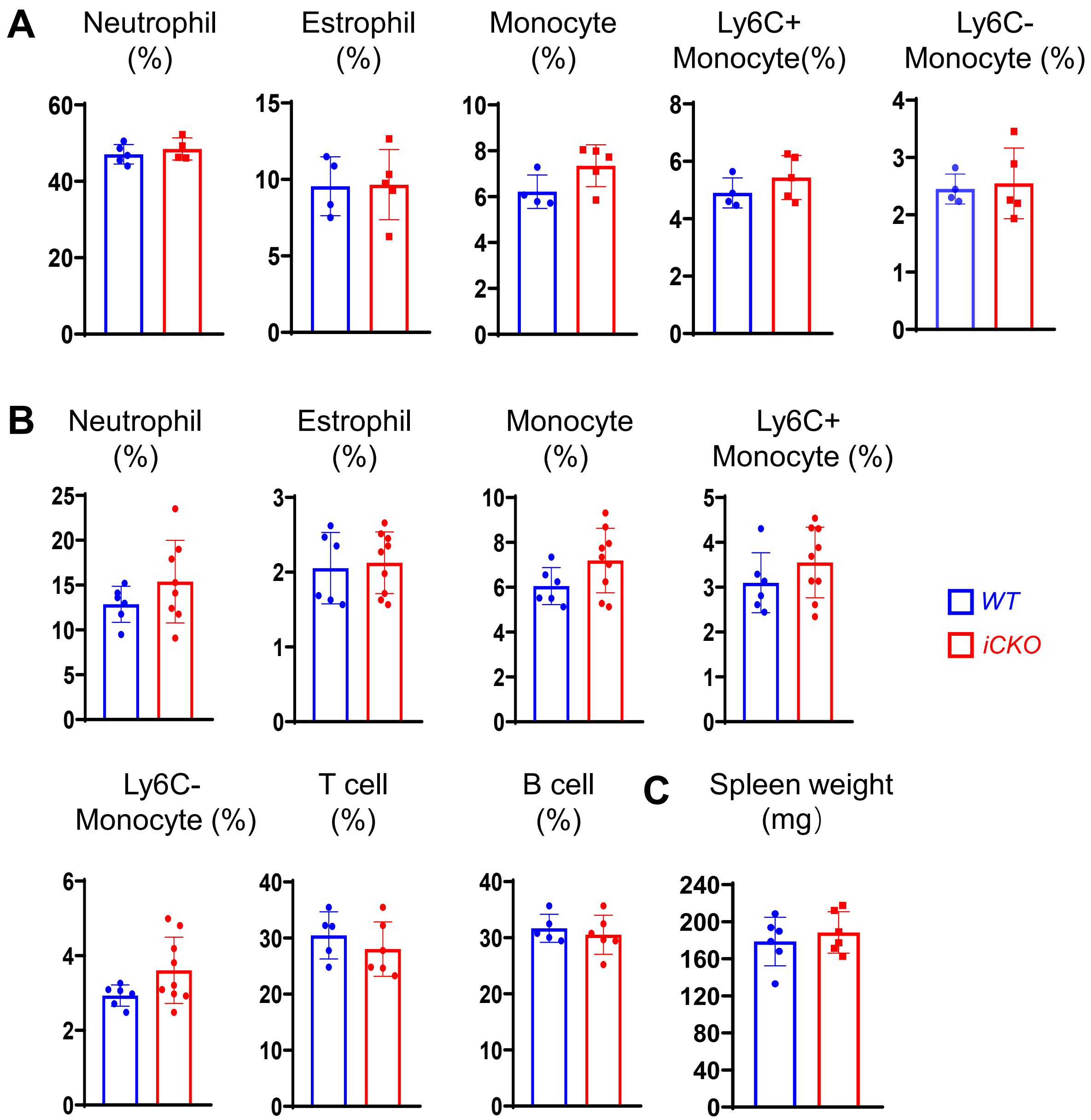

Figure S11

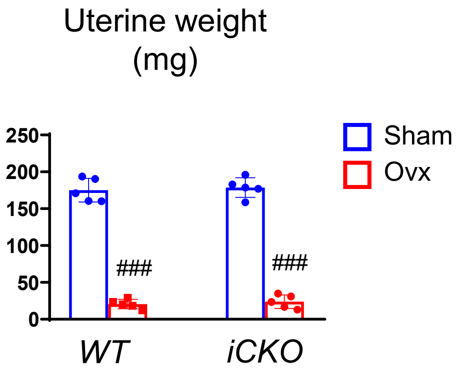

Figure S12

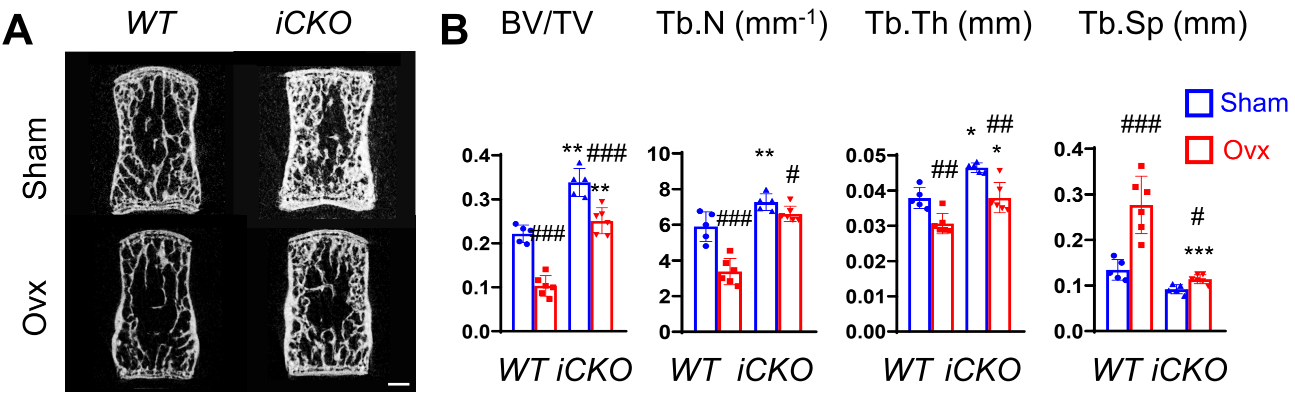

Figure S13

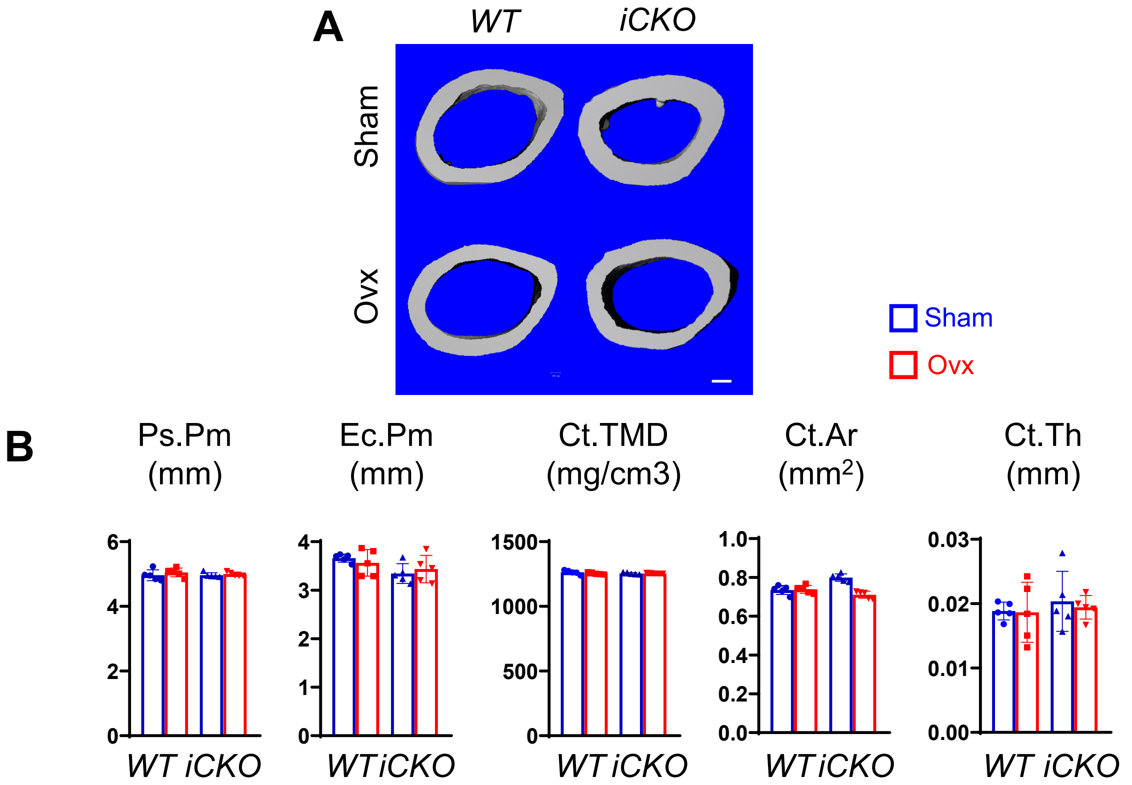

Figure S14

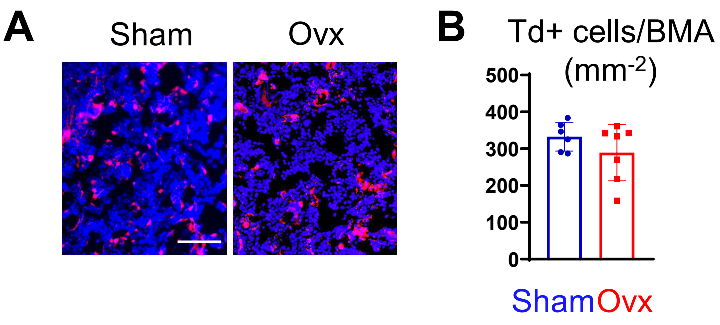

Figure S15

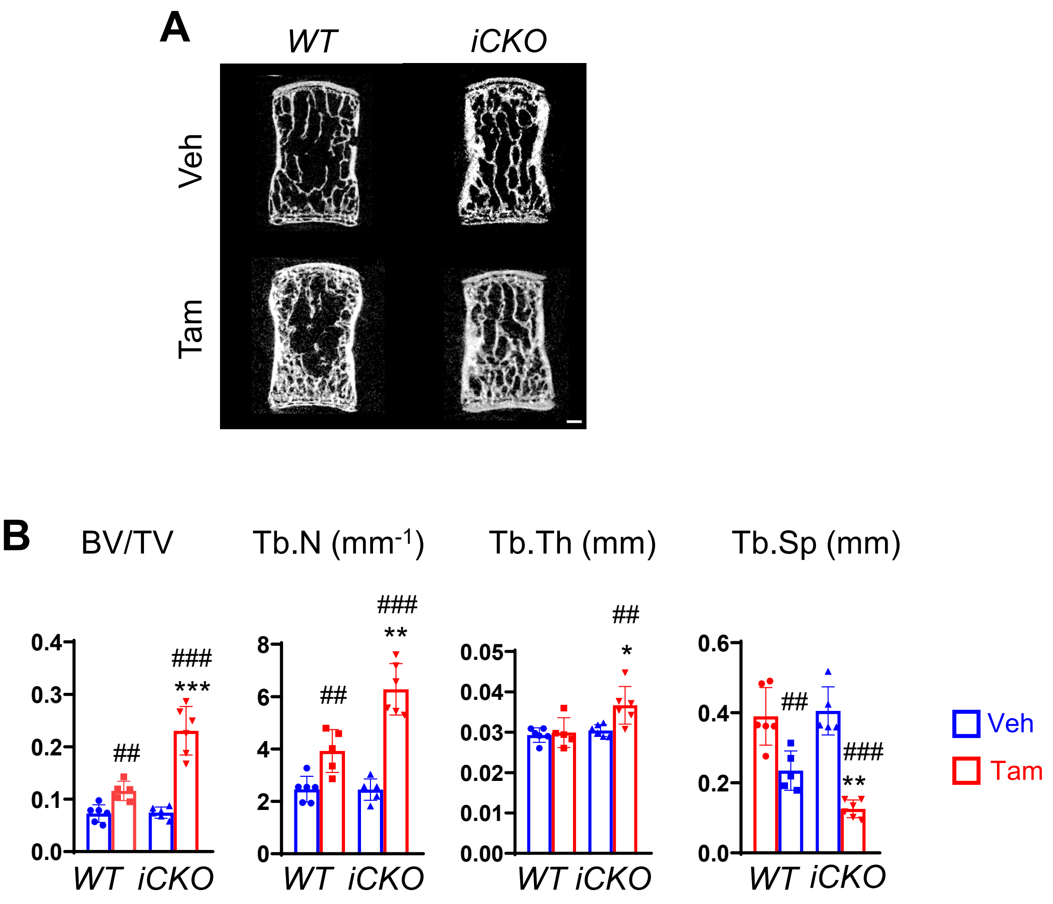

Supplement: Supplementary file 1 — Supplementary Materials [file 41413_2025_405_MOESM1_ESM.pdf]
